# Xiongshao Zhitong Recipe Attenuates Nitroglycerin-Induced Migraine-Like Behaviors *via* the Inhibition of Inflammation Mediated by Nitric Oxide Synthase

**DOI:** 10.3389/fphar.2022.920201

**Published:** 2022-07-19

**Authors:** Song Yang, Cong Chen, Xiaoyao Liu, Qianjun Kang, Quantao Ma, Pin Li, Yujie Hu, Jialin Li, Jian Gao, Ting Wang, Weiling Wang

**Affiliations:** ^1^ Beijing Research Institute of Chinese Medicine, Beijing University of Chinese Medicine, Beijing, China; ^2^ State Administration of Traditional Chinese Medicine Key Laboratory of Famous Doctors and Famous Prescriptions, Beijing, China; ^3^ National Medical Products Administration Key Laboratory for Research and Evaluation of Traditional Chinese, Beijing University of Chinese Medicine, Beijing, China; ^4^ School of Traditional Chinese Medicine, Beijing University of Chinese Medicine, Beijing, China

**Keywords:** Xiongshao Zhitong Recipe (XZR), migraine, NOS, NF-κB, SPR

## Abstract

Migraine is a major cause of disability worldwide, particularly in young adults and middle-aged women. Xiongshao Zhitong Recipe (XZR) is a traditional Chinese medicine prescription used for treating migraine, but its bioactive components and therapeutic mechanisms remain unclear. We aimed to confirm the therapeutic effect of XZR on migraine and to determine the possible mechanism and bioactive components of XZR. Here, a sensitive UHPLC-LTQ-Orbitrap MS assay was carried out to analyze the ingredients of XZR, and a total of 62 components were identified, including coumarins, phenolic acids, phthalides, flavonoids, and terpenoids; among them, 15 components were identified in the serum samples after XZR treatment. We established a rat model of migraine *via* nitroglycerin (NTG) injection. The *in vivo* experiments demonstrated that XZR attenuated allodynia and photophobia in rats with NTG-induced migraine, and XZR also demonstrated analgesic effects. XZR reversed the abnormal levels of nitric oxide, 5-hydroxytryptamine (5-HT), calcitonin gene-related peptide (CGRP), and substance P (SP) to normal levels. XZR also downregulated inflammatory reactions, including mast cell degranulation and serum IL-1β, IL-6, and TNF-α levels. In terms of mechanism, we revealed that XZR treated NTG-induced migraine through the inhibition of neuronal nitric oxide synthase (nNOS) and inducible nitric oxide synthase (iNOS) expression in both the trigeminal nucleus caudalis (TNC) and periaqueductal gray matter (PAG), as well as the total NOS enzyme activity, which regulated the NF-κB signaling pathway. Additionally, imperatorin and xanthotoxin, two major ingredients of XZR, showed a high binding affinity to nNOS (Gly468-Leu616). *In vitro*, XZR, imperatorin, and xanthotoxin inhibited the nNOS expression and the NF-κB signaling pathway in lipopolysaccharide (LPS)-stimulated PC12 cells. In conclusion, we demonstrated the therapeutic effects of XZR and provided evidence that XZR played a critical anti-inflammatory role by suppressing NOS and NF-κB signaling pathway activation. Imperatorin and xanthotoxin were potential bioactive components of XZR. The findings from this study supported that XZR was a candidate herbal drug for migraine therapy.

## Introduction

Migraine is a complex disorder characterized by recurrent disabling attacks of headache accompanied by nausea, vomiting, and paroxysmal neurovascular dysfunction (Raggi et al., 2022). The Global Burden of Diseases, Injuries, and Risk Factors Study 2016 (GBD 2016) showed that migraine was one of the leading causes of disability worldwide, especially among young adults and middle-aged women (Collaborators, 2018). The average morbidity and lifetime prevalence of migraine were 13.2% and 19%, respectively (Victor et al., 2010; Arroyo-Quiroz et al., 2014).

Although the mechanisms underlying migraine remain poorly understood, several main potential mechanisms have been suggested by researchers, including the activation of meningeal afferents, neuropeptide release, abnormal cranial vasodilation, neurogenic inflammation, and central pain sensitization (Edvinsson and Haanes, 2021). Much evidence supports that nitric oxide (NO) plays an important role in triggering migraine (Thomsen and Olesen, 2001). The synthesis of NO is catalyzed by nitric oxide synthase (NOS), which oxidizes a nitrogen atom in the guanidine group at the end of L-arginine (L-Arg) (Yuan et al., 2021). Through the NO-cyclic guanosine monophosphate (cGMP) pathway, NO can induce the initial phase of migraine headache associated with cerebral vasodilation and then trigger the delayed phase of migraine pain by stimulating the release of inflammatory neuropeptides, which results in sterile neurogenic inflammation and the sensitization of the perivascular nociceptors in the trigeminovascular system that promote migraine attacks (Reuter et al., 2001; Guo, 2017). NO also induces neuronal NOS (nNOS), which can be considered a significant marker of the sensitization of the trigeminal system (Spekker et al., 2021). Studies have also shown that the nuclear factor-kappa B (NF-κB) pathway plays an important role in the neurogenic inflammation of migraines. Following inflammatory injury, the NF-κB p65 subunit can be transferred to the nucleus and bind to specific DNA sequences, thereby initiating gene transcription and inducing the expression of multiple cytokines, including tumor necrosis factor-α (TNF-α), interleukin-1β (IL-1β), and interleukin-6 (IL-6) (Lai et al., 2019; Moisset et al., 2021).

Current treatments for migraine include general analgesics, such as nonsteroidal anti-inflammatory drugs (NSAIDs); specific painkillers, such as serotonin receptor agonists (such as triptans and 5-HT1B and 5-HT1D inhibitors); calcitonin gene-related peptide (CGRP) receptor antagonists [anti-CGRP monoclonal antibodies (mAbs)]; and selective CGRP receptor inhibitors (Silberstein, 2004). However, the adverse reactions of NSAIDs and triptans, including dizziness, nasopharyngitis, medication-overuse headache, and vascular risks, increase the difficulties of migraine treatment (Syed, 2016). Although anti-CGRP monoclonal antibodies effectively control migraines, a case series of probable migraine-related stroke, systemic inflammatory disorders, polyarthralgia, and reversible cerebral vasoconstriction syndrome following CGRP inhibition have been reported (Assas, 2021). Therefore, it is necessary to seek potential therapeutics for migraine.

Traditional Chinese medicine (TCM) has been used in the clinical treatment of migraine for many years (Li et al., 2011; Huang et al., 2020). Xiongshao Zhitong Recipe (XZR) is often used in the clinic for treating headaches due to wind–phlegm–blood stasis. A clinical study revealed that XZR shows a variety of desirable pharmacological effects on migraines, for instance, supporting Qi, promoting blood circulation, dispelling wind, and relieving pain (Su et al., 2016). However, the active ingredients and mechanism of XZR in treating migraine remain unknown. In this study, we established a migraine model with nitroglycerin (NTG) and evaluated the effects of XZR on migraine. In addition, we developed a surface plasmon resonance (SPR)-based high-throughput screening platform. With this platform and UHPLC-LTQ-Orbitrap MS, Western blotting, and immunofluorescence, we eventually determined the chemical composition of XZR and its possible mechanism of action in the treatment of migraine, and we preliminarily confirmed the active components in XZR with the NOS inhibitory activity.

## Materials and Methods

### Chemicals and Reagents

XZR comprises eight botanical drugs: Conioselinum anthriscoides “Chuanxiong” [*Apiaceae*] (No. 20200728), *Paeonia lactiflora* Pall. (No. 20200614), *Angelica dahurica* (Hoffm.) Benth. & Hook.f. ex Franch. & Sav. (No. 20200526), *Salvia miltiorrhiza* Bunge (No. 20200511), *Brassica juncea* (L.) Czern (No. 20200528), *Smilax glabra* Roxb (No. 20200611), *Beauveria bassiana* (Bals.) Vuillant (No. 20200212), and *Xanthium strumarium* subsp. strumarium (No. 20191128). Botanical drugs were provided by Bencao Fangyuan Group Co., Ltd. (Chongqing, China) and identified by Prof. Xiangri Li, Beijing University of Chinese Medicine. The samples of XZR (No. 20210111) were deposited in the Beijing Research Institute of Chinese Medicine, Beijing University of Chinese Medicine.

Rizatriptan was purchased from Hubei Ouly Pharmaceutical Co., Ltd. (Hubei, China). NTG injections were purchased from Beijing Yimin Pharmaceutical (China, 1 mg/ml). An NO assay kit (No. 13-2-1) and NOS activity assay kit (No. 14-1) were purchased from Nanjing Jiancheng Bioengineering Institute (Nanjing, China). 5-Hydroxytryptamine (5-HT) (No. CEA808Ge), TNF-α (No. SEA133Ra), and IL-1β (No. SEA563Ra) enzyme-linked immunosorbent assay (ELISA) kits and recombinant nitric oxide synthase 1 (NOS1) (RPA815Ra02) were purchased from Cloud-Clone Corp. (Wuhan, China). The IL-6 ELISA kit was from BioLegend (San Diego, CA, No. 437107). The CGRP ELISA kit was purchased from Bertin Bioreagent (France, No. 5482). The substance P (SP) ELISA kit was purchased from Cayman (United States, No. 583751). nNOS (C7D7) rabbit mAb (No. 4231), NeuN (E4M5P) mouse mAb (No. 94403), and the NF-κB Pathway Sampler Kit (No. 9936) were purchased from Cell Signaling Technology (United States). The anti-iNOS antibody was purchased from Abcam (United States, No. 3523). Neutral balsam (No. G8590) and toluidine blue O (No. G3670) were purchased from Beijing Solarbio Science Technology Group Co., Ltd. (Beijing, China).

### Preparation of Xiongshao Zhitong Recipe Samples

Botanical drugs were weighed in accordance with the proportions of XZR used in the clinic. Eight volumes of pure water were added. The extraction was performed 2 times for 1.5 h each time. After the first extraction, the filtrate was collected, and then six volumes of pure water were added for the second extraction. The combined extracts were filtered and concentrated at 80°C for 9 h under reduced pressure. After vacuum drying, 287.62 g of dry powder was obtained. The drug–extract ratio of XZR was 18.20%.

The intermediate dose of XZR (the equivalent clinical dose) used in rat experiments was calculated with the below formula: intermediate dose of XZR = 70.01 g/day × 18.20% × 6/70 kg.

The clinical raw drug dosage was 70.01 g/person/day, and the drug–extract ratio of XZR was 18.20%. On the basis of an average adult body weight of 70 kg, according to the body surface area (BSA) normalization method (Rockville et al., 2005; Nair and Jacob, 2016; Heinrich et al., 2020), the equivalent dose of human and rats was about 6.

The low dose was half of the equivalent clinical dose, while the high dose was twice the equivalent clinical dose. Accordingly, the three doses of XZR were 0.55, 1.09, and 2.18 g/kg/day, and the dose of rizatriptan was 0.857 mg/kg/day (the equivalent clinical dose).

### UHPLC-LTQ-Orbitrap MS Analysis of the Main Xiongshao Zhitong Recipe Components and Serum Components

The dried extract (0.2 g, 65 mesh) was accurately weighed and extracted by infusion with 50 ml of 80% methanol for 30 min. The extracted solution was filtered through a 0.22 μm nylon membrane filter before injection for the analysis of XZR components.

A total of 12 Sprague–Dawley male rats, weighing 250 ± 20 g (age of 6 weeks), were maintained for 12 h with no food but freely available water before treatment administration. The rats were randomly divided into two groups: the normal control group and the XZR group (*n* = 6). The rats in the XZR group were administered the XZR water extract at a dose of 7.644 g/kg, 7 times the equivalent clinical dose. The same volume of water was administered to the rats in the normal control group. Blood samples were obtained by the retro-orbital puncture at 0, 15, 30, and 60 min after XZR administration. After centrifugation for 15 min at 3,500 rpm, serum samples were acquired, and the mixed serum samples from the same group were purified in solid phase extraction (SPE) microcolumns for further analysis.

The identification of chemical constituents in XZR and the serum was performed with the UHPLC-LTQ-Orbitrap MS method. Chromatographic separation was performed on a Dionex Ultimate 3000 UHPLC Plus Focused Ultra High-Performance Liquid Chromatography System (Thermo Scientific, Santa Clara, CA, United States) comprising a UPLC pump, a DAD detector, scanning from 200 to 800 nm, and a cooling autosampler. The chromatographic conditions were as follows: column: ACQUITY UPLC BEH C18 (1.7 µm, 2.1 mm × 100 mm); solvent system: acetonitrile (A) and water containing 0.1% formic acid (B); gradient elution: 0–30.0 min, 5%–85% A; 30.1–35.0 min, 5% A; flow rate: 0.3 ml/min; injection volume: 10 μl; column temperature: 30°C. MS analysis was performed using an LTQ-OrbitrapXL hybrid mass spectrometer (Thermo Fisher Scientific) fitted with a HESI source and operated in negative and positive ion modes, with a mass range of 150–1,500 and a high resolution set at 30,000 using the normal scan rate. The data-dependent MS/MS events were always performed on the most intense ions detected in full-scan MS. The MS/MS isolation width was 1 amu. Nitrogen was used as the sheath gas, and helium served as the collision gas. The key optimized ESI-MSP parameters were as follows: source temperature: 300.0°C; source voltage: 4 kV; sheath gas (nitrogen): 50 L/min; auxiliary gas flow: 10 arb; capillary voltage: 25 V; and tube lens: 110.0 V. Data were collected and analyzed with Xcalibur 2.1 software (Thermo Fisher Scientific). Three batches of XZR were used to identify the concentrations of paeoniflorin and salvianolic acid B (Supplementary Material).

### Animals

The 60 specific pathogen-free (SPF) adult male Sprague-Dawley rats (200 ± 20 g) used in the study were provided by Beijing Vital River Laboratory Animal Technique Co., Ltd. (Beijing, China). The animals were kept in the Experimental Animal Center at the Beijing University of Chinese Medicine (Beijing, China) at 22 ± 2°C on a 12-h/12-h light/dark cycle. The animals were given regular feed and free access to water. All studies were strictly performed in accordance with the international ethical guidelines and related ethical regulations of the Beijing University of Chinese Medicine (No. BUCM-4-2021020101-1009).

### Migraine Model Induced by Nitroglycerin

The rats were randomly divided into six groups: the control group, NTG control group (NTG group, 10 mg/kg), rizatriptan group (rizatriptan, 0.0857 mg/ml), XZR low-dose group (XZR-L group, 0.55 g/kg), XZR intermediate-dose group (XZR-M group, 1.09 g/kg), and XZR high-dose group (XZR-H group, 2.18 g/kg). Rats in the rizatriptan group, XZR-L group, XZR-M group, and XZR-H group were intragastrically administered the respective drugs once per day for seven consecutive days. All rats, except those in the control group, were subcutaneously injected with NTG 30 min after the last treatment (Zhang et al., 2017). Rats in the control group were injected with an equivalent volume of distilled water.

### Behavioral Test

The frequency of head scratching was measured with a video camera (DSC-WX9, China). Briefly, a video camera was placed away from the cubicle in positions facing the subject. Thirty minutes after NTG injection, all rats were acclimatized to the cubicles for 5 min, and then the scratching behaviors of the rats were recorded for 1.5 h. Scratching behaviors were quantified based on the observations of the defined events [for details, see Chanda et al. (2013)] in a blinded manner and counted by two colleagues from a digital video.

### Mechanical Threshold Test

Thirty minutes after NTG injection, the mechanical threshold was tested as described previously (Gautam and Ramanathan, 2021). Briefly, the facial and plantar surfaces of the rat were stimulated with electronic von Frey filaments. A series of filaments (0–60 g) were applied on the facial and plantar surfaces with pressure causing the filament to buckle and held for approximately 6–8 s. The average withdrawal reading of three trials was recorded as the final value.

### Light-Aversive Test

A light–dark test was used to evaluate light-aversive behavior 30 min after NTG injection (Bonnet et al., 2019). Briefly, rats were acclimatized for 5 min in a light-aversion chamber (25 cm × 25 cm × 40 cm) before testing. Light-aversive behavior was examined within 30 min after NTG injection. Rats were placed in the light zone of the light-aversion chamber, and data were collected for 30 min. Tests were administered at least 5 min apart.

### Toluidine Blue O Dyeing of Dural Mast Cells

The dura mater was isolated at 1.5 h after NTG injection on the 7th day of XZR administration. The isolated dura mater was placed on a glass slide and stained with toluidine blue for 2 min. The dura mater was washed with PBS three times and then fixed with ethanol (95%, 85%, and 75%). Images were taken at ×20 magnification by an Evos FL Auto 2 (Thermo Fisher Scientific, America). Mast cells with inhomogeneous staining, pale cells, and cells with disfigured borders surrounding the positively stained granules were classified as degranulated (Loewendorf et al., 2016). The rate of degranulation was calculated as the number of degranulated cells to the total number of cells.

### Biochemical Determination

All rats were anesthetized with 4% pentobarbital sodium at 1.5 h after model establishment. Blood samples were collected from the abdominal aorta to determine plasma 5-HT and serum IL-1β, IL-6, SP, and CGRP levels with the ELISA method, while the level of NO in serum was detected with an NO biochemical kit (Nanjing Jiancheng Institute of Biological Engineering, Nanjing, China) by the colorimetric method. The brain tissues of these rats were used to test the TNF-α level by ELISA.

### Western Blot Analysis

The rat trigeminal nucleus caudalis (TNC) and periaqueductal gray matter (PAG) were collected at 1.5 h after model establishment. Tissues were lysed in the RIPA lysis buffer. The protein concentration was determined with a bicinchoninic acid (BCA) kit (Beyotime, China). The protein samples (50 μg) were separated on sodium dodecyl sulfate–polyacrylamide gel electrophoresis (SDS–PAGE) gels and transferred onto polyvinylidene fluoride (PVDF) membranes (Bio-Rad, United States). The membranes were blocked with 5% skim milk for 2 h at room temperature. Then, the membranes were incubated with primary antibodies, namely, anti-iNOS (1:500), anti-NF-κB (1:1,000), anti-nNOS (1:1,000), anti-Iκbα (1:1,000), and anti-Ikkβ (1:1,000), at 4°C overnight. The next day, the membranes were incubated with the secondary antibody for 1 h at room temperature. Then, the protein bands were detected with electrochemiluminescence (ECL) and analyzed with an Amersham Imager 680 (Cytiva, United States).

### Immunofluorescence Staining

The rat TCN and PAG were fixed in 4% paraformaldehyde 1.5 h after model establishment, dehydrated with sucrose solution, and coated with ETC. Tissues were cut into 5 μm sections with a Leica CM1900 cryostat. The TCN and PAG sections were washed three times with PBS, blocked in 2% goat serum, and incubated with the following primary antibodies: anti-nNOS (1:50), anti-iNOS (1:50), and anti-NeuN (1:200) overnight at 4°C. On the next day, the samples were incubated with the corresponding secondary antibodies at room temperature for 1 h without light, and then DAPI (10 μg/ml) was added for 10 min to stain the nuclei. Images were obtained using Evos FL Auto2. The relative area and the mean fluorescence intensity were analyzed using ImageJ software.

### Affinity Measurement

Biacore T200 was used to detect the specific binding between the main constituents of XZR absorbed in the blood and recombinant nNOS (Gly468-Leu616). Biacore T200 (GE Healthcare) was used to measure the binding affinities (Huang et al., 2021). nNOS (Gly468–Leu616) was diluted in sodium acetate solution (pH 5.0) to a final concentration of 50 μg/ml. The solution of nNOS (Gly468–Leu616) was immobilized on a CM5 sensor chip (GE Healthcare) by amine coupling to reach target densities of 12,000 resonance units (RUs). Immobilized nNOS (Gly468–Leu616) was used to capture the chemical compound. The running buffer contained PBS-T (10 mM sodium phosphate, 150 mM NaCl, 0.005% Tween-20, pH 7.4) and 1% DMSO. Then, eight concentrations of each molecule (0, 1.56, 3.125, 6.25, 12.5, 25, 50, and 100 µM) were injected at a flow rate of 30 μl/min and 25°C. Blank immobilization was performed on one of the sensor chip surfaces for the correction of the binding response. The protein binding time and dissociation time were both 120 s. Sensorgrams were analyzed using Biacore T200 Evaluation version 3.2.1 (Cytiva).

### Cell Culture

PC12 cells were purchased from the Cell Resource Center of Shanghai Institutes for Biological Sciences, Chinese Academy of Sciences (Shanghai, China), cultured in 1640 medium supplemented with 10% FBS and 1% penicillin/streptomycin and incubated at 37°C with 5% CO_2_. PC12 cells were seeded into 6-well plates at a density of 2 × 10^4^ cells/ml. After 24 h of culture, the culture medium was replaced with a serum-free medium and cultured for another 12 h. Then, the cells were treated with L-NAME (1 mM), XZR (25, 50 and 100 μg/ml), imperatorin (IMP) (12.5, 25, and 50 μM), and xanthotoxin (XAN) (12.5, 25, and 50 μM) for 48 h, and an *in vitro* inflammation model was induced by lipopolysaccharide (LPS) (1 μg/ml) intervention for 0.5 h. The PC12 cells were harvested for Western blot detection.

### Statistical Analysis

All data are shown as the mean ± standard deviation (SD). All statistical analyses were performed by SPSS 20.0 statistical software. Statistical differences were determined with a one-way analysis of variance (ANOVA), and a least significant difference (LSD) post hoc test was used for comparing the mean values. The level of significant difference was set at *p* < 0.05.

## Results

### Chemical Composition of Xiongshao Zhitong Recipe

**FIGURE 1:**
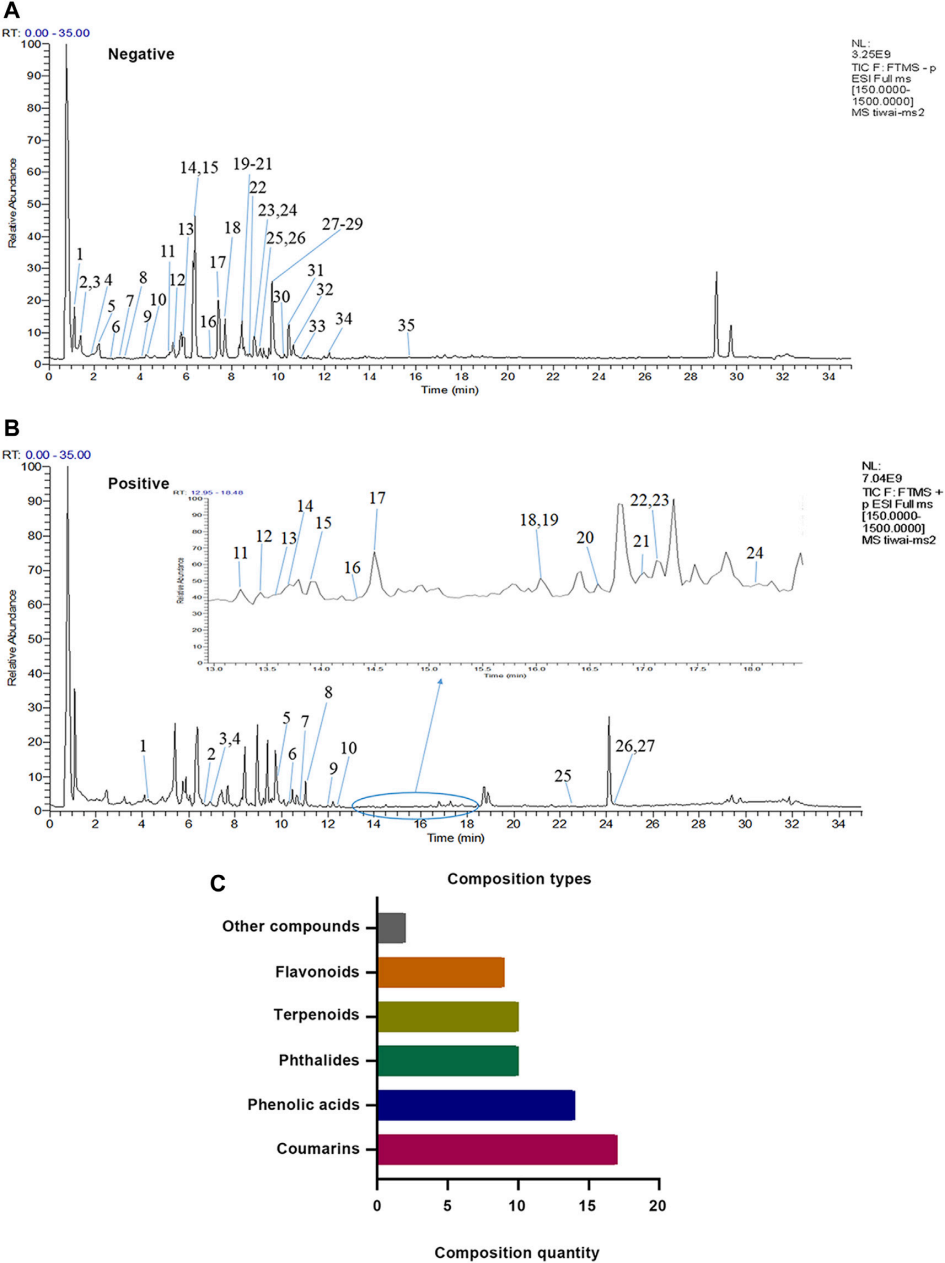
Identification results of the main chemical components of XZR by UHPLC-LTQ-Orbitrap MS. **(A)** Total ion flow diagram of XZR in anion mode (details of Nos. 1–35 are listed in Supplementary Table S1). **(B)** Total ion flow diagram of XZR in the positive ion mode (details of Nos. 1–27 are listed in Supplementary Table S2). **(C)** Identification of the main components in XZR. XZR, Xiongshao Zhitong Recipe.

A total of 62 compounds were identified in XZR, including 27 components in the positive ion mode and 35 components in the negative ion mode (Figures 1A,B; Supplementary Tables S1, S2). The 62 identified chemical components included 17 coumarins, 14 phenolic acids, 10 phthalides, 10 terpenoids, 9 flavonoids, and 2 other components (Figure 1C). The retention times, molecular formulas, and MS2 fragment ions of the identified components in XZR are summarized in Supplementary Tables S1, S2.

### Xiongshao Zhitong Recipe Improved the Migraine-Like Behaviors of Rats With Nitroglycerin-Induced Migraine

Subcutaneous injection of NTG was used to establish a migraine rat model and evaluate the efficacy of XZR. After the subcutaneous injection of NTG, the typical behaviors of frequent head scratching, cage climbing, and photophobia began to appear in rats of the NTG group within 3–5 min and lasted for at least 2 h.

**FIGURE 2:**
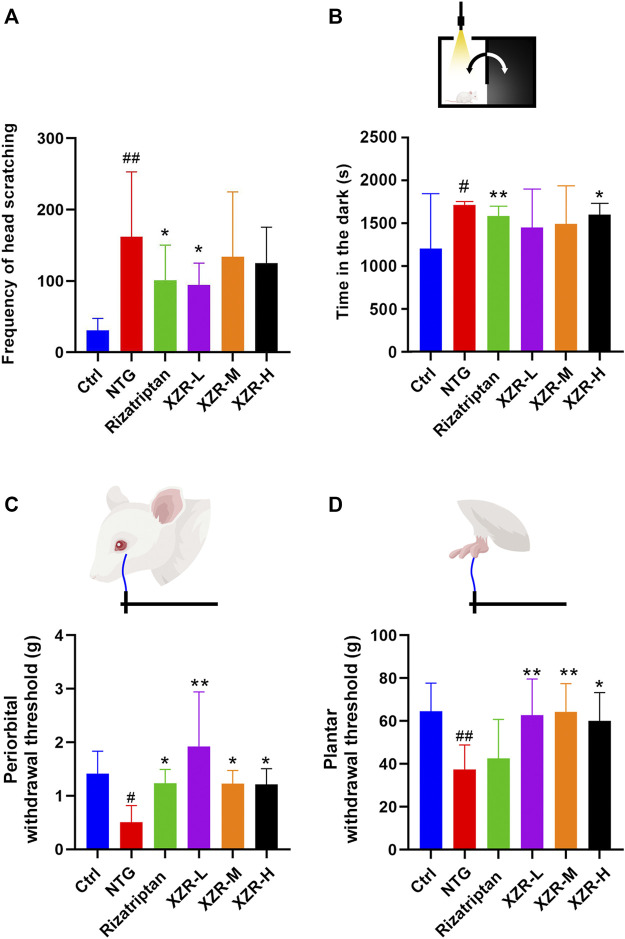
XZR improved migraine-like behavior in the NTG-induced migraine rat model. **(A)** Frequency of head scratching. **(B)** Time in the dark chamber. **(C)** Periorbital withdrawal threshold. **(D)** Plantar withdrawal threshold. Data are presented as the mean ± standard deviation. ^#^ *p* < 0.05, ^##^ *p* < 0.01 versus control group, *p < 0.05, **p < 0.01 versus NTG group, *n* = 7–10. XZR, Xiongshao Zhitong Recipe; NTG, nitroglycerin.

The behavioral characteristic of head scratching indicated successful establishment of a migraine rat model (Li, et al., 2011; Gao et al., 2014). As shown in Figure 2A, rats in the control group occasionally scratched their heads within 90 min of behavioral monitoring. The number of head scratches was nearly 30. Compared with that in the control group, the number of head scratches in the NTG group was markedly increased (161.80 ± 91.15, *p* < 0.01). The rizatriptan (101.00 ± 49.43, *p <* 0.0*5*) and XZR-L (94.50 ± 30.61, *p <* 0.05) groups showed significantly fewer head scratches than the NTG group, while the XZR-M (134.00 ± 90.77) and XZR-H (124.90 ± 50.55) groups also showed fewer head scratches than the NTG group, but the difference was not significant.

In Figures 2C,D, NTG injection evoked strong extracephalic tactile allodynia, as the periorbital and plantar withdrawal thresholds (periorbital allodynia and plantar allodynia) were 0.51 ± 0.31 g and 37.37 ± 11.53 g, respectively, which were much lower than those in the control group. Rizatriptan and XZR administration significantly attenuated NTG-induced periorbital allodynia, as the animals had enhanced mean periorbital withdrawal thresholds (rizatriptan group 1.23 ± 0.26 g, *p <* 0.05; XZR-L group 1.92 ± 1.02 g, *p* < 0.01; XZR-M and XZR-H groups, 1.23 ± 0.25 g and 1.22 ± 0.29 g, respectively, *p* < 0.05). Furthermore, XZR treatments significantly attenuated NTG-induced plantar allodynia, as the mean plantar withdrawal thresholds in rats were enhanced (XZR-L and XZR-M groups 62.73 ± 16.88 g and 64.23 ± 13.26 g, respectively, *p* < 0.01; XZR-H group 60.07 ± 13.22 g, *p* < 0.05). XZR also exhibited an analgesic effect in the acetic-acid-induced writhing model, as represented by a significant decrease in the frequency of writhing (Supplementary Figure S1).

In addition to pain, the disabling symptoms of migraine often include photophobia (Bonnet, et al., 2019). The light-aversive behavior (photophobia) of rats after NTG injection was evaluated using the light–dark transition test. As shown in Figure 2B, 30 min after NTG injection, the rats in the NTG group spent 1.4-fold more time in the dark chamber than those in the control group. Rats in the rizatriptan (1584.00 ± 111.80 s) and XZR-H (1602 ± 126.50 s) groups showed no signs of photophobia, as the animals spent significantly less time in the dark chamber than those in the NTG group (1713.00 ± 38.42 s).

Altogether, these data indicated that XZR effectively attenuated NTG-induced cephalic and extracephalic tactile allodynia in rats with migraine.

### Xiongshao Zhitong Recipe Regulated Migraine Mediators in the Nitroglycerin-Induced Migraine Rat Model

The pathological response of migraine is closely related to the release of related neuropeptides, such as CGRP and SP, and neurotransmitters, such as 5-HT. ELISA was performed to evaluate the levels of these migraine mediators in serum and plasma.

**FIGURE 3:**
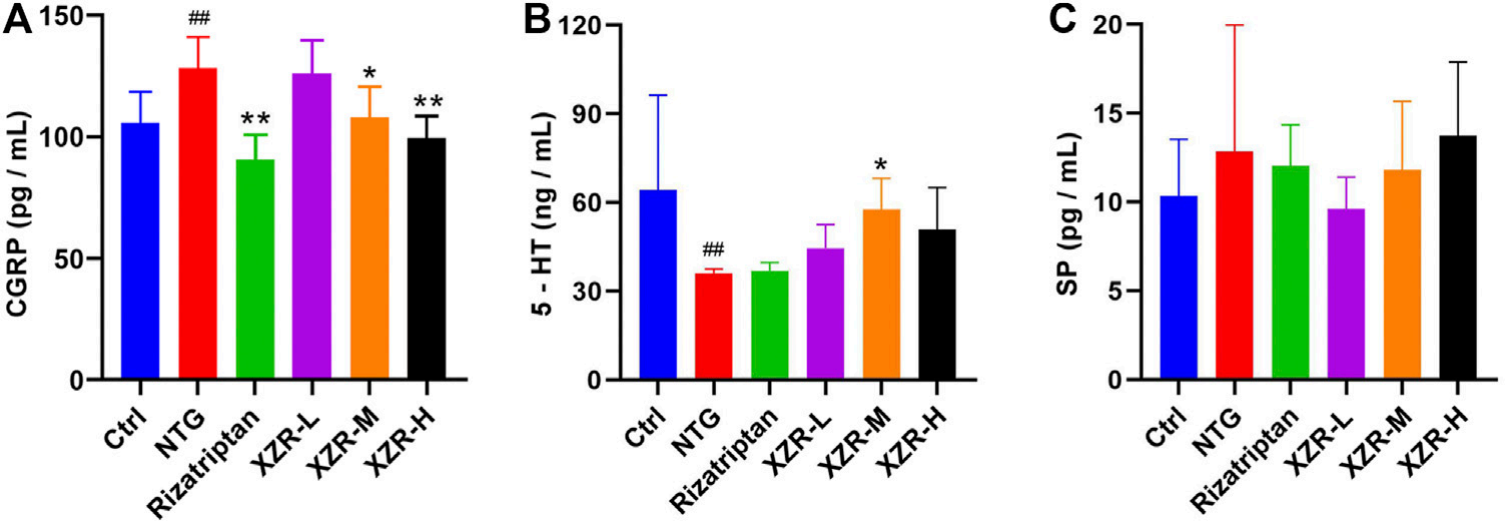
XZR regulated neuropeptide and neurotransmitter levels in rats with NTG-induced migraine. **(A)** CGRP in serum, *n* = 6, **(B)** 5-HT in plasma, *n* = 6, and **(C)** SP in serum, *n* = 5. Data are presented as the mean ± standard deviation. ^##^ *p* < 0.01 versus the control group, *p < 0.05, **p < 0.01 versus the NTG group. XZR, Xiongshao Zhitong Recipe; CGRP, calcitonin gene-related peptide; 5-HT, 5-hydroxytryptamine; SP, substance P; NTG, nitroglycerin.

As shown in Figure 3A, the level of serum CGRP was significantly increased in the NTG group (127.66 ± 12.98 pg/ml) compared with that in the control group (104.93 ± 13.52 pg/ml), while compared with the NTG group, the levels of serum CGRP in the XZR groups (XZR-L group 124.53 ± 15.49 pg/ml, *p* > 0.05; XZR-M group 107.33 ± 12.75 pg/ml, *p* < 0.05; and XZR-H group 98.27 ± 9.93 pg/ml, *p* < 0.01) were significantly decreased in a dose-dependent manner. Rats in the XZR-H group even showed a return of the high level of serum CGRP to normal levels. Furthermore, there was a significant decrease in serum CGRP levels after rizatriptan treatment (89.50 ± 11.18, *p* < 0.01).

The plasma 5-HT concentration (36.19 ± 1.34 ng/ml, *p* < 0.01) was significantly decreased in the NTG group compared with that in the control group (64.25 ± 32.15 ng/ml). After XZR treatments, plasma 5-HT concentrations were increased, and the optimal effect was seen in the XZR-M group (57.71 ± 10.57 ng/ml, *p* < 0.05) (Figure 3B).

Although the levels of SP did not significantly change after the interventions, there was a clear downward trend in the XZR-L group (Figure 3C).

### Xiongshao Zhitong Recipe Reduced Markers of Inflammation in the Nitroglycerin-Induced Migraine Rat Model

**FIGURE 4:**
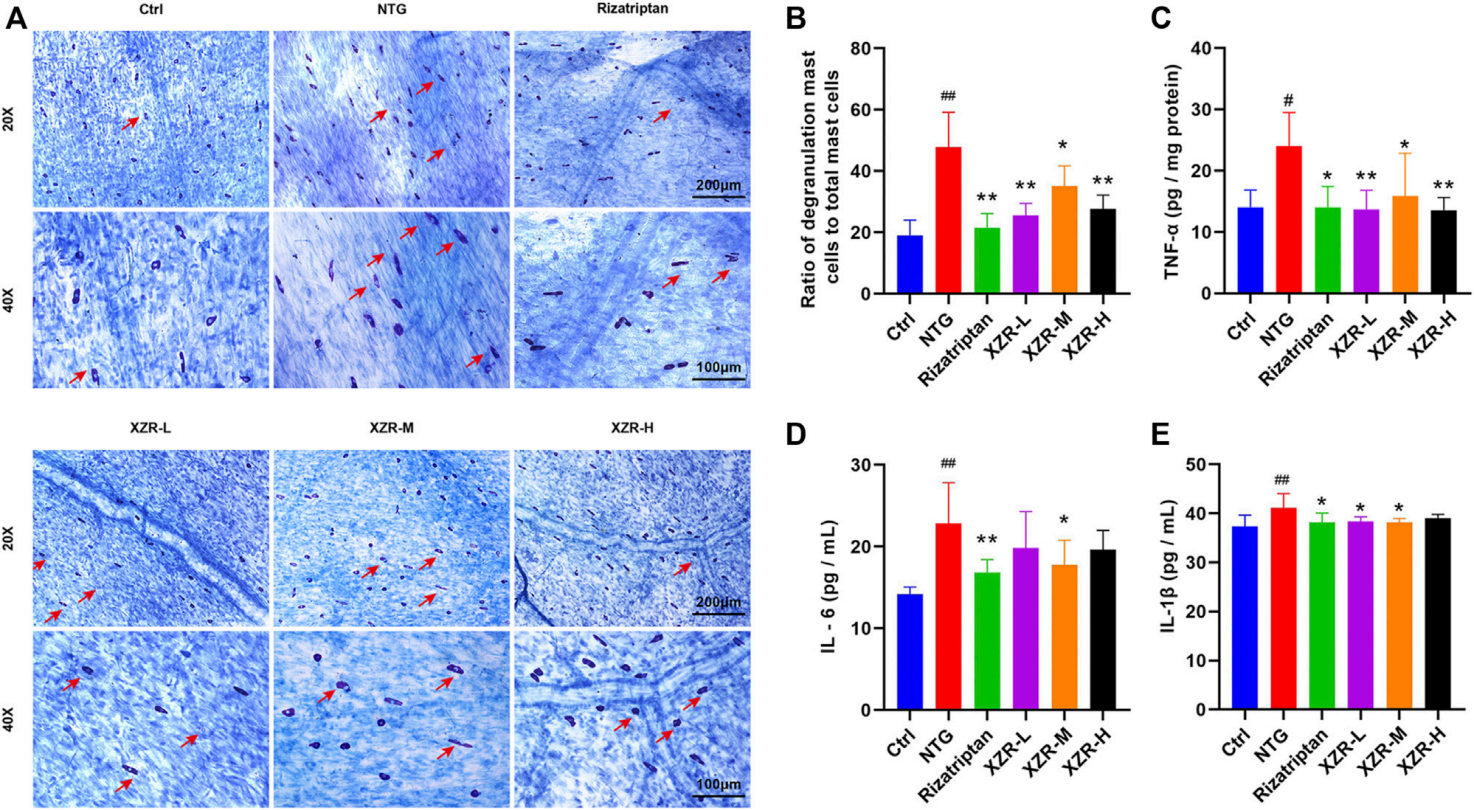
XZR reduced the inflammation of rats with NTG-induced migraine. **(A)** Toluidine blue staining of mast cells in the dura (original magnification ×20 and ×40). Scale bars = 200 μm (×20) and 100 μm (×40). The arrow indicates degranulated mast cells. **(B)** Ratio of degranulated mast cells to total mast cells. **(C)** TNF-α in brain and **(D)** IL-6 and **(E)** IL-1β in serum. Data are presented as the mean ± standard deviation, *n* = 5–6. ^#^ *p* < 0.05, ^##^ *p* < 0.01 versus the control group, *p < 0.05, **p < 0.01 versus the NTG group. XZR, Xiongshao Zhitong Recipe; NTG, nitroglycerin.

The degranulation of dural mast cells indicates local inflammation, nociceptive afferent activation of TG neurons, and vasodilation (Irmak et al., 2019), suggesting that activated dural mast cells may mediate headache. Mast cell degranulation of the dura was detected in our experiment. Toluidine blue staining showed that mast cells in the control group were long, spindle-shaped, and dark purple. Mast cells in the NTG group were irregular in shape, enlarged in volume, and released granular substances, which were significantly alleviated after XZR treatment (Figure 4A). As shown in Figure 4B, the percentage of degranulated mast cells in rats in the NTG group (47.73% ± 11.41%) was much higher than that in the control group (19.06% ± 4.97%, *p* < 0.01). Compared with the NTG group, the percentages of degranulated mast cells in the dura in the rizatriptan (21.52% ± 4.65%), XZR-L (25.52% ± 3.94%), and XZR-H groups (27.66% ± 4.48%) were obviously decreased (*p* < 0.01).

To further investigate the effect of XZR on inflammation, the secretion of cytokines, including TNF-α, IL-1β, and IL-6, was also detected. As shown in Figures 4C–E, compared with the control group, the level of TNF-α (24.05 ± 5.42 pg/mg protein) in the midbrain and the concentrations of IL-1β (41.11 ± 2.92 pg/ml) and IL-6 (22.80 ± 5.02 pg/ml) in serum were significantly increased in the NTG group (*p* < 0.01). Rizatriptan almost decreased the TNF-α (14.04 ± 3.41 pg/mg protein), IL-6 (16.83 ± 1.59 pg/ml), and IL-1β (38.21 ± 1.86 pg/ml) concentrations to normal levels. Moreover, intermediate-dose XZR had a similar beneficial effect, as it reversed the high levels of TNF-α (15.91 ± 6.93 pg/mg protein), IL-1β (38.20 ± 0.76 pg/ml), and IL-6 (17.76 ± 3.02 pg/ml) in rats with migraine (*p* < 0.05). Both the XZR-L (13.73 ± 3.07 pg/mg protein) and XZR-H (13.57 ± 2.06 pg/mg protein) groups showed significantly decreased TNF-α levels (*p* < 0.01), and the XZR-L group (38.87 ± 0.93 pg/ml) showed obviously reduced levels of IL-1β, compared with the NTG group (*p* < 0.05).

### Xiongshao Zhitong Recipe Inhibited Nitric Oxide Synthase-Mediated Nitric Oxide Production in Rats With Nitroglycerin-Induced Migraine

**FIGURE 5:**
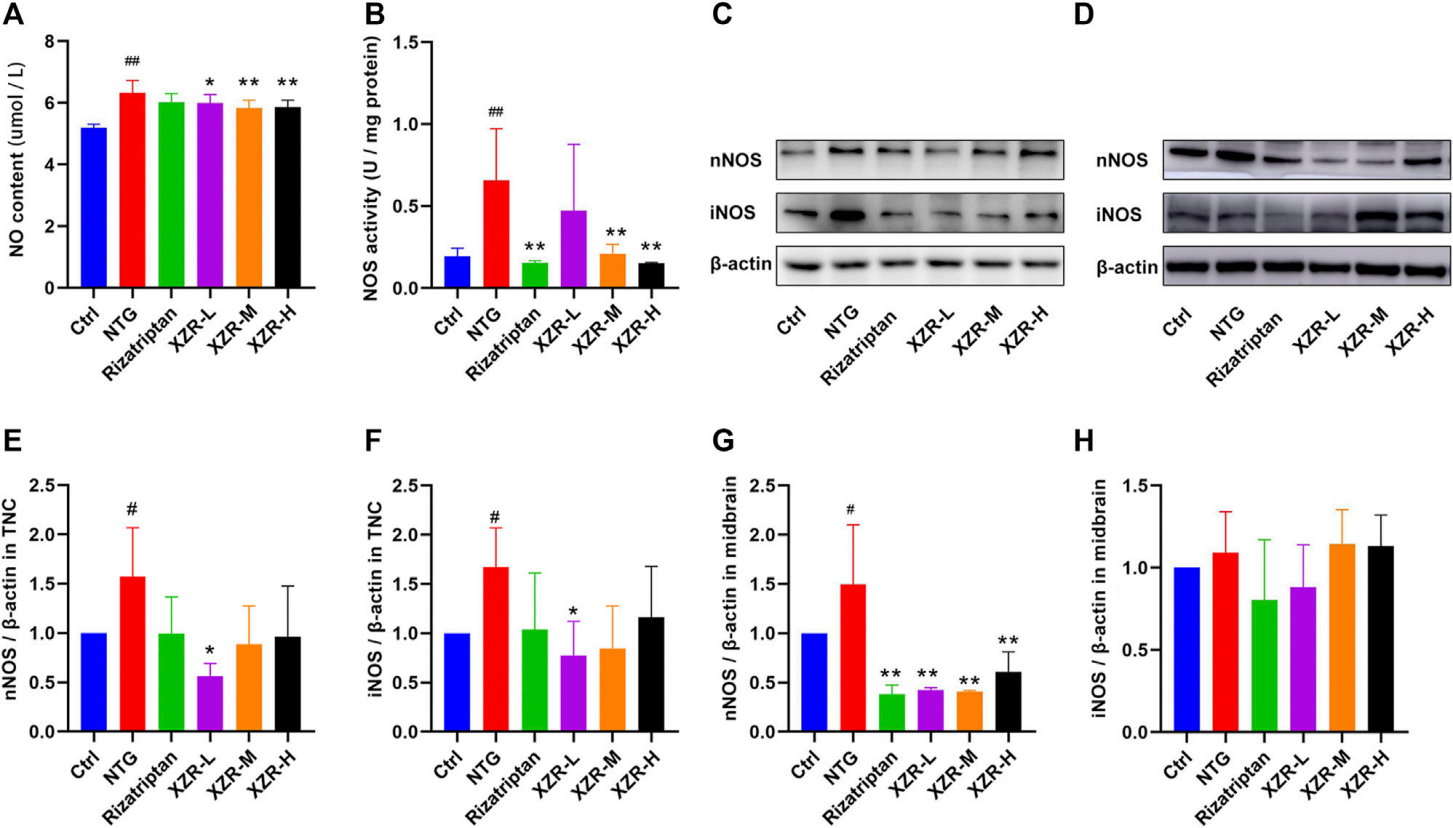
XZR inhibited the NOS-mediated production of NO in rats with NTG-induced migraine. **(A)** NO level and **(B)** NOS activity in midbrain, *n* = 6. **(C,D)** Relative expression levels of nNOS and iNOS in the TNC and midbrain were determined by Western blotting. **(E,F)** Relative expression levels of nNOS and iNOS in the TNC. **(G,H)** Relative expression levels of nNOS and iNOS in the midbrain. Data are presented as the mean ± standard deviation, *n* = 3. ^#^ *p* < 0.05, ^##^ *p* < 0.01 versus control group, *p < 0.05, **p < 0.01 versus the NTG group. XZR, Xiongshao Zhitong Recipe; TNC, trigeminal nucleus caudalis; NTG, nitroglycerin.

As an endogenous gaseous signaling molecule, NO regulation is altered in migraine pathogenesis, and NO is endogenously produced in the body by NOS. NO and NOS are clearly important regulators of migraine (Pradhan et al., 2018). ELISA was performed to evaluate the NO level and NOS activity in plasma. As shown in Figure 5A, the level of plasma NO in the NTG group (6.33 ± 0.40 μM) was significantly increased compared with that in the control group (5.19 ± 0.12 μM, *p* < 0.01), and XZR (XZR-L 5.99 ± 0.28 μM, *p* < 0.05; XZR-M 5.84 ± 0.25 μM, *p* < 0.01; XZR-H 5.86 ± 0.23 μM, *p* < 0.01) significantly reduced the NO levels. More importantly, the NOS activity in the NTG group (0.66 ± 0.32, *p* < 0.01) was much higher, nearly 3-fold, than that in the control group (0.19 ± 0.05) (Figure 5B). Compared with treatment with NTG, the NOS activity was obviously decreased after treatment with rizatriptan (0.15 ± 0.01, *p* < 0.01) and the intermediate (0.21 ± 0.06, *p* < 0.01) and high (0.15 ± 0.01, *p* < 0.01) doses of XZR, which was consistent with the NO results.

In addition, Western blot analysis was performed to observe the expression of nNOS and iNOS in the TNC and midbrain. In the TNC, the results showed a significantly increased expression of nNOS (1.57 ± 0.50, *p < 0.05*) and iNOS (1.67 ± 0.40, *p* < 0.05) in the NTG group (Figures 5C,E,F), reduced nNOS expression levels in the XZR-L group (0.56 ± 0.13, *p* < 0.05), and the return of iNOS expression to normal levels in the XZR-L group (0.77 ± 0.35, *p* < 0.05). In the midbrain, the abnormal expression of nNOS was observed only in the NTG group (1.50 ± 0.61), and all treatments, including rizatriptan (0.38 ± 0.09, *p* < 0.01) and low (0.43 ± 0.02, *p* < 0.01), intermediate (0.41 ± 0.01, *p* < 0.01) and high (0.61 ± 0.20, *p* < 0.01) doses of XZR, downregulated nNOS expression levels compared with those in the NTG group (Figures 5D,G,H).

**FIGURE 6:**
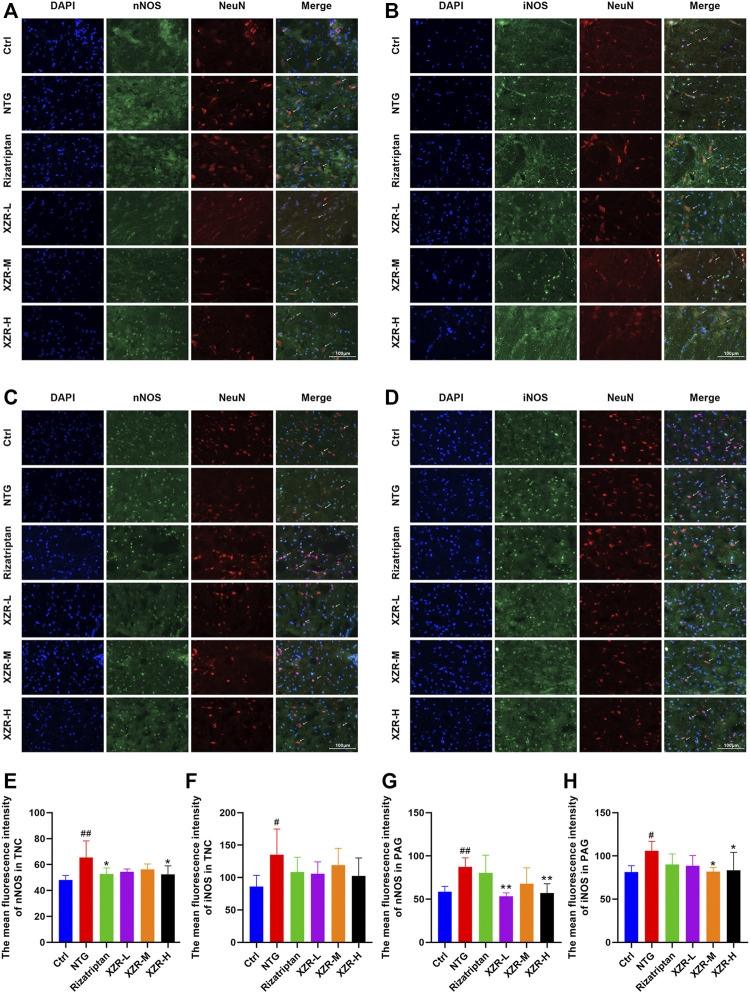
XZR reduced the NOS expression in the TNC and PAG. **(A,B)** Immunofluorescence of nNOS and iNOS in the TNC and **(C,D)** PAG. Immunofluorescence double-label, DAPI (blue), nNOS and iNOS (green), and NeuN (red), scale bar = 100 μm. **(E,F)** The mean fluorescence intensity of nNOS and iNOS in the TNC. **(G,H)** The mean fluorescence intensity of nNOS and iNOS in the PAG. Data are presented as the mean ± standard deviation, *n* = 6. ^#^ *p* < 0.05, ^##^ *p* < 0.01 versus the control group, *p < 0.05, **p < 0.01 versus the NTG group. XZR, Xiongshao Zhitong Recipe; TNC, trigeminal nucleus caudalis; NTG, nitroglycerin; PAG, periaqueductal gray.

For further confirmation, the expression and localization of nNOS and iNOS in the rat spinal TNC and PAG were observed by immunofluorescence. The results of the immunofluorescence double-label experiments were shown in Figure 6. In the TNC, the fluorescence intensities of nNOS (65.36 ± 12.97, *p* < 0.01; Figures 6A,E) and iNOS (135.30 ± 39.62, *p* < 0.05; Figures 6B,F) were significantly increased in the NTG group compared with those in the control group, and the fluorescence intensity of nNOS was significantly decreased in the rizatriptan and XZR-H groups compared with that in the NTG group (52.83 ± 4.52 and 52.45 ± 6.56, respectively, *p* < 0.05). In the PAG, the fluorescence intensities of nNOS (87.26 ± 10.49, *p* < 0.01; Figures 6C,G) and iNOS (105.80 ± 11.16, *p* < 0.05; Figures 6D,H) were increased in the NTG group compared with those in the control group, while the fluorescence intensity of nNOS in the XZR-L and XZR-H groups (53.26 ± 4.06 and 57.01 ± 10.76, respectively, *p* < 0.01) was significantly decreased and the fluorescence intensity of iNOS in the XZR-M and XZR-H groups (81.86 ± 4.73 and 83.39 ± 20.85, respectively, *p* < 0.05) was decreased compared to that in the NTG group.

### Xiongshao Zhitong Recipe Inhibited Inflammation *via* the NF-κB Signaling Pathway

Accompanied by the abnormal expression of cytokines and NOS, the NF-κB signaling pathway is also involved in the neurogenic inflammation caused by NTG-induced migraine. Furthermore, Western blotting was performed to investigate the expression of proteins in the NF-κB signaling pathway, including IKKβ, IκBα, and NF-κB in the TNC and midbrain, and the effect of XZR.

**FIGURE 7:**
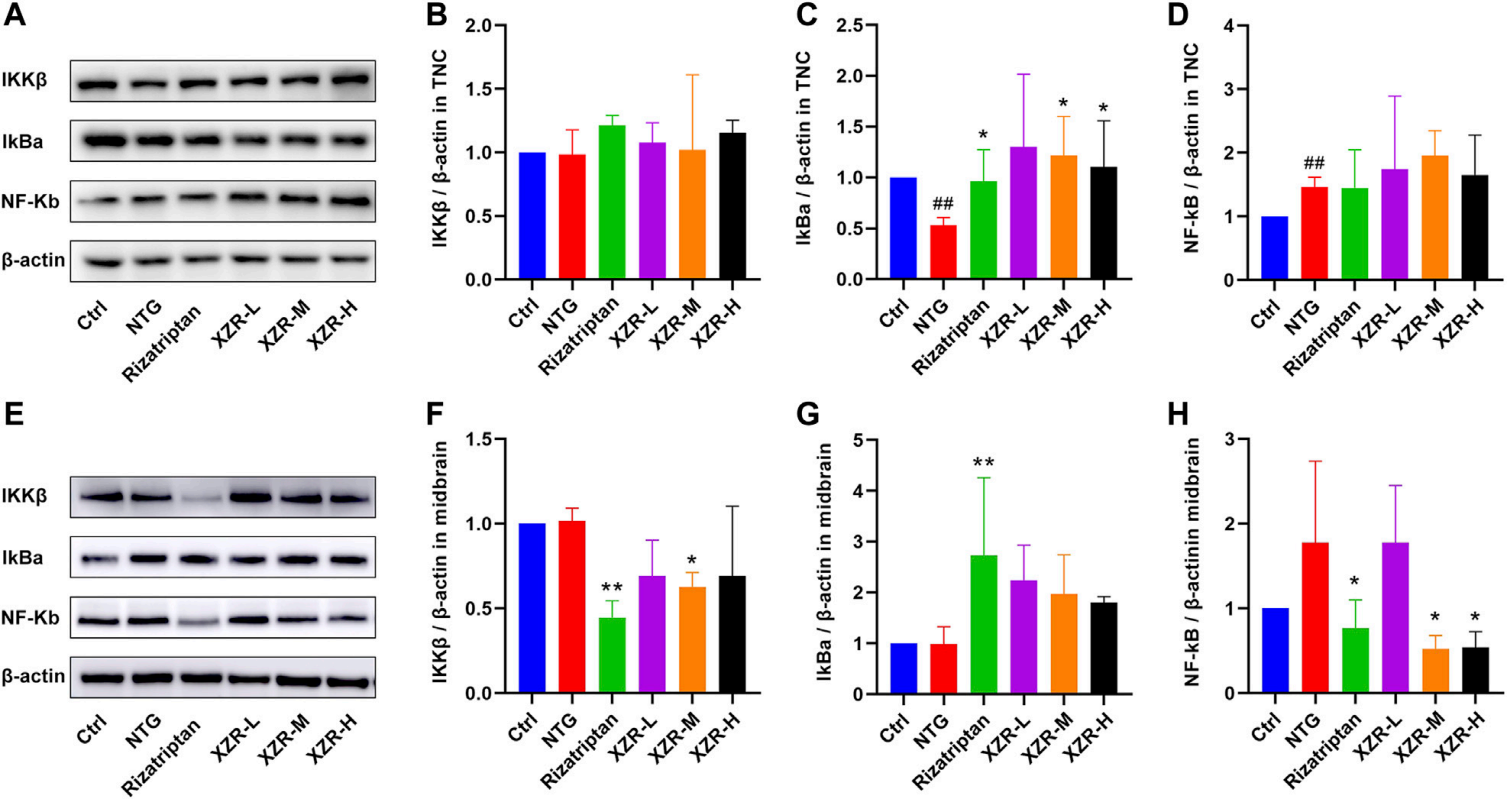
XZR inhibited inflammation *via* the NF-κB signaling pathway. **(A)** Representative Western blot images of IKKβ, IκBα, and NF-κB expression in the TNC. **(B)** Relative expression of IKKβ in the TNC. **(C)** Relative expression of IκBα in the TNC. **(D)** Relative expression of NF-κB in the TNC. **(E)** Representative Western blot images of IKKβ, IκBα, and NF-κB expression in the midbrain. **(F)** Relative expression of IKKβ in the midbrain. **(G)** Relative expression of IκBα in the midbrain. **(H)** Relative expression of NF-κB in the midbrain. Data are presented as the mean ± standard deviation, *n* = 3–5. ^##^ *p* < 0.01 versus the control group, *p < 0.05, **p < 0.01 versus the NTG group. XZR, Xiongshao Zhitong Recipe; NTG, nitroglycerin.

As shown in Figures 7A–D, in the TNC, the expression level of IκBα (0.53 ± 0.08, *p* < 0.01) was obviously reduced and that of NF-κB (1.46 ± 0.15, *p* < 0.01) was increased in the NTG group compared with that in the control group, while rizatriptan (0.96 ± 0.31, *p* < 0.05) and intermediate (1.22 ± 0.38, *p* < 0.05) and high (1.10 ± 0.46, *p* < 0.05) doses of XZR markedly increased the expression of IκBα.

In the midbrain (Figures 7E,F), the expression level of IKKβ in the rizatriptan (0.45 ± 0.10, *p* < 0.01) and XZR-M (0.63 ± 0.09, *p <* 0.05) groups was obviously reduced after treatment compared with that in the NTG group (1.02 ± 0.08). Figures 7E,G show that the IκBα expression in the rizatriptan group (2.73 ± 1.53, *p* < 0.01) was significantly increased, and low (2.2 ± 0.69), intermediate (1.97 ± 0.78), and high (1.80 ± 0.12) doses of XZR showed a tendency to increase the IκBα expression compared with that in the NTG group (0.99 ± 0.34). Figures 7E,H show that NF-κB expression in the rizatriptan (0.77 ± 0.33), XZR-M (0.52 ± 0.16), and XZR-H (0.54 ± 0.19) groups was obviously reduced after treatment compared with that in the NTG group (1.78 ± 0.96, *p <* 0.05).

### Potential Target Analysis of Xiongshao Zhitong Recipe in Treating Migraine

**FIGURE 8:**
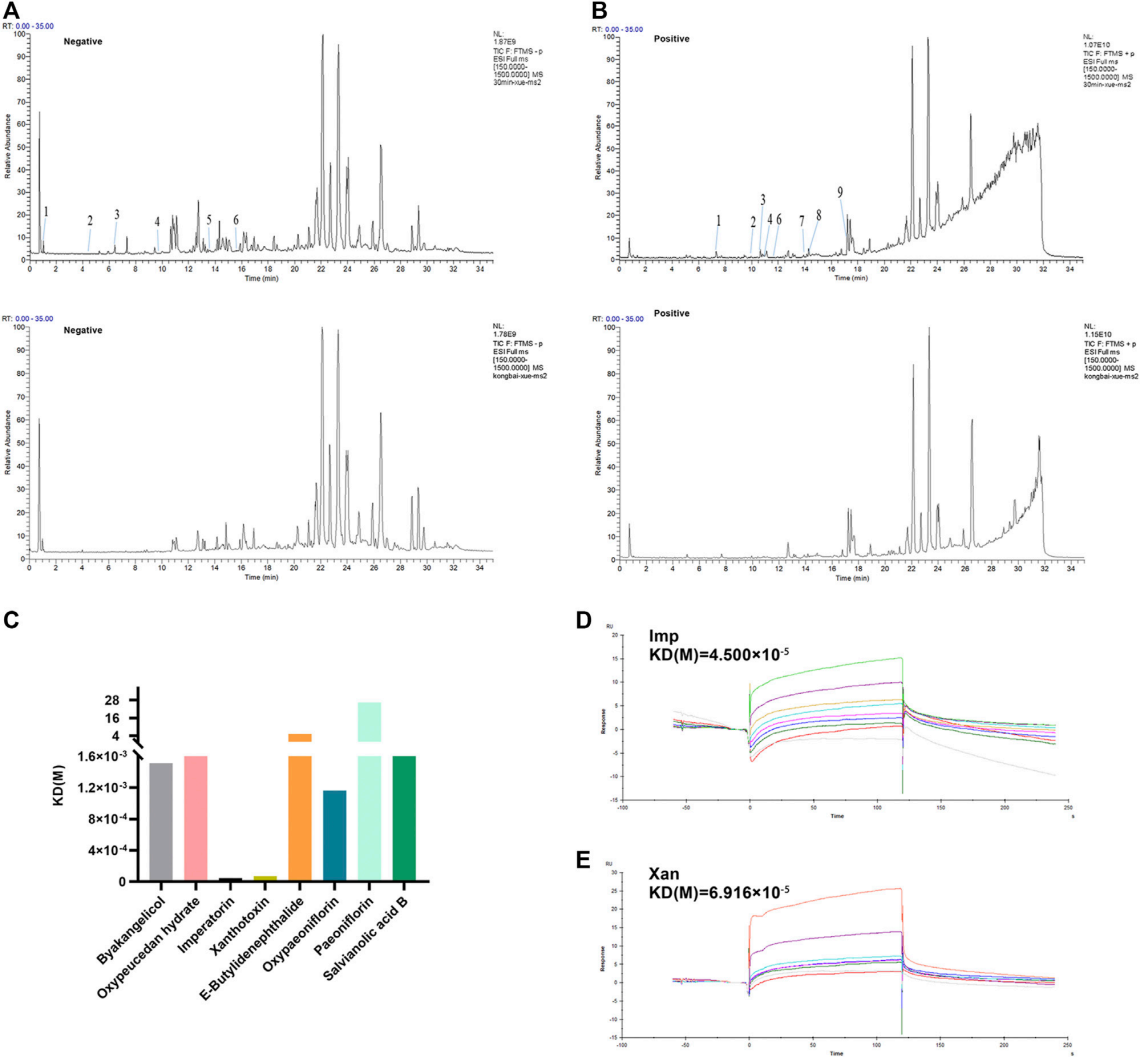
Analysis of candidate targets of XZR against migraine. **(A)** Chromatographic and mass spectral data of the chemical compounds of XZR in the serum analyzed by UHPLC-LTQ-Orbitrap MS (negative mode) (top: serum sample acquired after XZR administration; bottom: blank serum sample. The details of Nos. 1–6 are listed in Supplementary Tables S3. **(B)** Chromatographic and mass spectral data of the chemical compounds of XZR in the serum analyzed by UHPLC-LTQ-Orbitrap MS (positive mode) (top: serum sample acquired after XZR administration; bottom: blank serum sample. The details of Nos. 1–9 are listed in Supplementary Tables S4. **(C)** Biacore analysis of eight compounds identified in serum bound to nNOS (Gly468–Leu616). **(D)** Biacore analysis of IMP bound to nNOS (Gly468–Leu616). **(E)** Biacore analysis of XAN bound to nNOS (Gly468–Leu616). XZR, Xiongshao Zhitong Recipe; Imp, imperatorin; Xan, xanthotoxin.

Based on the above results, we provide strong evidence for a role of XZR in the treatment of migraine. However, the pharmacodynamic basis and molecular mechanism of XZR in its effect on migraine remain unclear. TCM prescriptions are characterized by multiple components, multiple targets, and multiple pathways. Therefore, the main constituents of XZR dissolved in serum were detected by the UHPLC-LTQ-Orbitrap MS. As shown in Figures 8A,B, a total of 15 components of XZR (including 7 coumarins, 3 phthalides, 3 terpenoids, and 2 phenolic acids) were identified in serum samples after XZR treatment—9 in the positive ion mode and 6 in the negative ion mode. The retention times, molecular formulas, and MS2 fragment ions of the identified exogenous ingredients of XZR are summarized in Supplementary Tables S3 and S4.

SPR is a novel and straightforward methodology used to study protein–compound interactions. Among the binding results between nNOS and the 15 constituents of XZR, imperatorin (Imp) and xanthotoxin (Xan) were found to directly bind to nNOS (Gly468–Leu616) in a concentration-dependent manner and showed a high affinity [K_D_ (Imp) = 0.45 μM, K_D_ (Xan) = 0.69 μM, respectively] (Figures 8C–E). However, the other components did not show such binding patterns.

### Imperatorin and Xanthotoxin Inhibited the Nitric Oxide Synthase/NF-κB Signaling Pathway *In Vitro*

**FIGURE 9:**
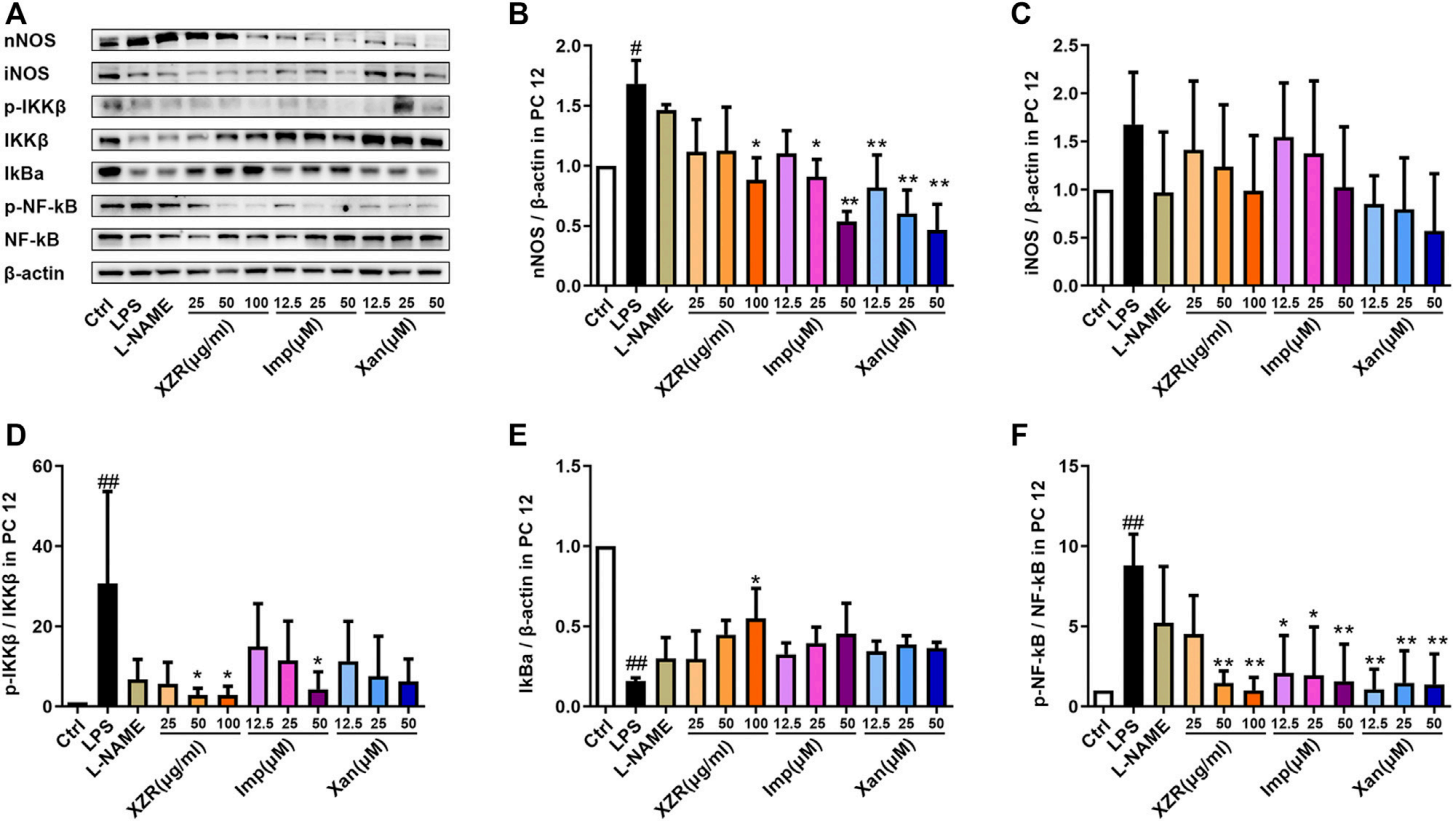
IMP and xanthotoxin inhibited the NOS/NF-κB signaling pathway *in vitro*. **(A)** Representative Western blot images of nNOS, iNOS, IKKβ, IκBα, and NF-κB expression in LPS-treated PC12 cells. **(B)** Relative expression of nNOS. **(C)** Relative expression of iNOS. **(D)** Relative expression of IKKβ. **(E)** Relative expression of IκBα. **(F)** Relative expression of NF-κB. Data are presented as the mean ± standard deviation, *n* = 3–5. ^#^ *p* < 0.05, ^##^ *p* < 0.01 versus the control group, *p < 0.05, **p < 0.01 versus the LPS group. LPS: lipopolysaccharide. IMP: imperatorin.

To validate the potential effect of imperatorin and xanthotoxin on the NOS/NF-κB signaling pathway, *in vitro* experiments were conducted. As shown in Figures 9A,B, nNOS expression was significantly higher in LPS-stimulated PC12 cells (1.68 ± 0.20) than in untreated cells (*p* < 0.05). Treatment with 25 μg/ml, 50 μg/ml, and 100 μg/ml XZR reduced nNOS expression. However, the differences were statistically significant only at high doses of XZR (0.88 ± 0.32, *p* < 0.05). As expected, both imperatorin and xanthotoxin inhibited the nNOS expression in a dose-dependent manner. Imperatorin (25 μM 0.91 ± 0.29, *p* < 0.05; 50 μM 0.54 ± 0.08, *p* < 0.01) and xanthotoxin (12.5 μM 0.82 ± 0.27, *p* < 0.01; 25 μM 0.60 ± 0.34, *p* < 0.01; 50 μM 0.47 ± 0.21, *p* < 0.01) significantly reduced the nNOS expression compared with that in the LPS group. The iNOS expression in LPS-stimulated PC12 cells (1.68 ± 0.54, Figures 9A,C) was nearly 1.7-fold higher than that in untreated cells, and XZR (100 μg/ml 0.99 ± 0.58) almost reversed the high level of iNOS in LPS-stimulated PC12 cells to normal levels. Interestingly, there was little effect of imperatorin on the iNOS expression, while xanthotoxin inhibited the iNOS expression in a dose-dependent manner.

Similarly, markers of the activated NF-κB signaling pathway were observed in the supernatant of LPS-stimulated PC12 cells. The expression of *p*-IKKβ/IKKβ (30.71 ± 22.95, *p* < 0.01) and *p*-NF-κB/NF-κB (8.80 ± 1.95, *p* < 0.01) was significantly increased, and the expression of IκBα (0.16 ± 0.02, *p* < 0.01) was significantly reduced by LPS (Figures 9D–F).

In particular, XZR at 50 μg/ml and 100 μg/ml significantly inhibited the NF-κB signaling pathway in LPS-stimulated PC12 cells (Figures 9D–F). XZR (50 μg/ml and 100 μg/ml) treatment significantly reduced the expression of *p*-IKKβ/IKKβ (2.85 ± 1.71, *p* < 0.05; 2.86 ± 2.09, *p* < 0.05) and *p*-NF-κB/NF-κB (1.48 ± 0.74, *p* < 0.01; 1.00 ± 0.41, *p* < 0.01), and XZR (100 μg/ml) upregulated IκBα (0.55 ± 0.19, *p* < 0.05) expression. Different dosages of imperatorin and xanthotoxin inhibited the NF-κB signaling pathway to varying degrees. Imperatorin (50 μM) downregulated *p*-IKKβ/IKKβ (2.85 ± 1.71, *p* < 0.05), and all doses of imperatorin (12.5 μM 2.11 ± 2.32, *p* < 0.05; 25 μM 1.95 ± 3.05, *p* < 0.05; 50 μM 1.59 ± 2.31, *p* < 0.01) and xanthotoxin (12.5 μM 1.08 ± 1.25, *p* < 0.01; 25 μM 1.48 ± 1.99, *p* < 0.01; 50 μM 1.38 ± 1.90, *p* < 0.01) downregulated the expression of *p*-NF-κB/NF-κB.

## Discussion

Migraine is a widespread neurological disorder that affects nearly one billion people worldwide (Collaborators, 2018). According to GBD 2016, migraine is the second leading cause of disability, with a higher disability rate than all other neurological disorders combined (Disease, 2017). Migraine imposes a heavy financial burden on patients and countries due to high healthcare costs, work absences, and reduced productivity. Researchers are interested in developing therapies for migraine. XZR is one of the TCM recipes for treating headaches due to wind–phlegm–blood stasis. A clinical study revealed that XZR has a variety of desirable pharmacological effects on migraines (Su, et al., 2016). However, the effective components and mechanism of action of XZR in the treatment of migraine remain unknown. Furthermore, due to the complex chemical components of XZR, it is difficult to elucidate the potential active compounds and precise pharmacological mechanisms involved in treating migraine and improving inflammatory conditions. We conducted a systemic study to evaluate the bioactive components and pharmacological mechanisms of XZR in the treatment of migraine.

This study has several highlights: 1) XZR, a Chinese herbal decoction, was effective in improving migraine-like behavior, including frequent head scratching, photophobia, and hyperalgesia; 2) XZR inhibited inflammation mediated by the NF-κB signaling pathway and the expression of CGRP in NTG-induced migraine; 3) XZR inhibited the NF-κB signaling pathway activation by inhibiting NOS in NTG-induced migraine; and 4) imperatorin and xanthotoxin interacted with nNOS and inhibited the NF-κB signaling pathway, suggesting that imperatorin and xanthotoxin might be effective substances in XZR.

Because of the complexity and variability in the botanical drugs of TCM, we strictly explored the process of extraction, purification, concentration, and granulation and carried out strict quality control at each step, including the property analysis, identification, and inspection of each herb and content determination of index components. As shown in Supplementary Figures S2, Table S5, the concentrations of paeoniflorin and salvianolic acid B in different batches of XZR were nearly 10.78 mg/g XZR extract and 16.46 mg/g XZR extract, respectively. More importantly, two batches of XZR were applied in animal experiments (Figure 2; Supplementary Figure S3), and the frequency of head scratching was similar. These data suggested that our extraction methods ensured that the different batches had good repeatability and reproducibility.

NTG, an NO donor compound, has been strongly implicated in the pathological mechanisms of migraine (Giesen et al., 2020). NTG administration was used to mimic the episodic migraine condition (Akerman et al., 2019). The reliability of the NTG-induced migraine model resides in its ability to reproduce headache attacks with features of spontaneous migraine attacks. In the present study, NTG (10 mg/kg) successfully provoked migraine that resulted in mechanical hyperalgesia and migraine-like behavior, including red ears, frequent head scratching, cage climbing, and photophobia, which was consistent with the clinical features of migraine, including head pain accompanied by nausea, vomiting, photophobia, and phonophobia (Dodick, 2018).

It was reported that women are three times more likely to suffer from migraines than men (Labastida-Ramirez et al., 2019). We evaluated the therapeutic effect of XZR on migraine in both sexes of animals in our previous experiment. The results showed that XZR showed a more obvious therapeutic effect on male rats than female rats (Supplementary Figure S3). Male rats showed fewer individual differences than female rats. Therefore, we chose male rats for this research. Utilizing an NTG-induced migraine model, we demonstrated that XZR could improve migraine symptoms. The number of head scratches and the time in the dark chamber were significantly decreased in XZR-treated rats compared with rats with NTG-induced migraine. XZR increased periorbital von Frey thresholds and paw sensory thresholds. Moreover, XZR reduced the writhing frequency in the acetic-acid-induced writhing model. All these data suggest that XZR exhibits excellent therapeutic effects on migraine.

The dose (e.g., g/day) per day and kg body weight used in the *in vivo* studies must be of therapeutic relevance. It is important to fully discuss the reason for using excessively high doses in the field of ethnopharmacology (Heinrich et al., 2020). As highlighted by Heinrich, medicinal plants require strict dose risk control to be of value in guiding clinical practice (Heinrich, 2013). XZR was a recipe of modified Sanpian decoction, which recorded in “Bian zheng lu” by Shiduo Chen in Qing dynasty. Conioselinum anthriscoides “Chuanxiong” [*Apiaceae*] (No. 20200728), *P. lactiflora* Pall. (No. 20200614), *A. dahurica* (Hoffm.) Benth. & Hook.f. ex Franch. & Sav. (No. 20200526), and *B. juncea* (L.) and Czern (No. 20200528) were the main drugs of Sanpian decoction. Sanpian decoction has a long history of therapy migraine (Xu et al., 2020). A meta-analysis has revealed the efficacy and safety of Sanpian decoction on migraine (Wu et al., 2020). The results showed that Sanpian decoction significantly improved the clinical efficacy [relative risk (RR) 4.19, 95% confidence intervals (CIs) 2.91–6.04, *p* < 0.00001; RR 1.29, 95% CI 1.09–1.54, *p* = 0.003 separately) and there were minor side effects related to Sanpian decoction, which were well tolerated. The dose of Sanpian decoction used in clinics was 94 g/per day for adult. According to the clinical practical experience of Sanpian decoction, XZR consisted of 15 g Conioselinum anthriscoides “Chuanxiong” [*Apiaceae*] (No. 20200728), 20 g *P. lactiflora* Pall. (No. 20200614), 10 g *A. dahurica* (Hoffm.) Benth. & Hook.f. ex Franch. & Sav. (No. 20200526), 15 g *S. miltiorrhiza* Bunge (No. 20200511), 10 g *B. juncea* (L.) Czern (No. 20200528), 20 g *S. glabra* Roxb (No. 20200611), 10 g *B. bassiana* (Bals.) Vuillant (No. 20200212), and 6 g *X. strumarium* subsp. strumarium (No. 20191128). The dosage of each herb in XZR complies with the provisions of Chinese Pharmacopoeia 2020. In clinics, the dose of XZR was 70 g/per day for adults. And the real-world research revealed that XZR improved migraine with little side effect (Su, et al., 2016). In summary, the effective dose of XZR was 70 g/per day for adults.

The rat dose in our study was calculated based on the body surface area and the corresponding clinically prescribed dose for a 70 kg human body [70.01 g (raw herbs)/70 kg/day] (Zhang et al., 2021). Therefore, the dosage of XZR-middle is almost equivalent to the human clinical dose, and the low dose is half of the equivalent clinical dose, while the high dose is double the equivalent clinical dose. The doses of XZR extract used in rats in this study were also consistent with that in most previous studies, that is, the dosage of decoction in rodents reached crude drug g/kg/day (Jiang et al., 2020; Zhang et al., 2021). It is worth emphasizing that the quantitative analysis of bioactive compounds in XZR and studies on the low dose levels assessing the therapeutic effect of XZR decoction warrant further exploration in the future.

In our study, XZR at a low concentration showed the optimal pharmacological effect on the frequency of head scratching as well as head and plantar withdrawal. The low dose of XZR was half of the equivalent clinical dose, while the high dose was double the equivalent clinical dose. The intermediate dose of XZR was the equivalent clinical dose. Usually, the equivalent clinical dose of this TCM showed the best pharmacological effect. We also wondered why the low dose of XZR exhibited the best pharmacological effect. We considered that there are two possible reasons: 1) large individual differences. As shown in Figure 2A, the frequency of head scratching in rats in the NTG group was 161.80 ± 91.15, while the frequencies of head scratching were 134.00 ± 90.77 and 124.90 ± 50.55 after intermediate (XZR-M) and high (XZR-H) doses of XZR, respectively. The SD was high in these three groups. However, the XZR-M and XZR-H groups showed an obvious decreasing trend in the frequency of head scratching. Increasing the sample size and decreasing the standard deviation may be necessary in future research. 2) TCM prescriptions are characteristically multicomponent, multitarget, and multipath compounds for the comprehensive treatment of diseases. This phenomenon is commonly encountered in the efficacy evaluation of TCM prescriptions. In past decades, many efforts have been made to reveal the dose–effect relationships of TCM prescriptions. It was difficult to accurately evaluate the efficacy of TCM prescriptions because they are often mixtures of multiple components. In TCM, a prescription characteristically contains more than one herbal drug and multiple components for multiple treatment targets (Zha et al., 2015). TCM prescription dose–effect relationships cannot be described as simply as chemical drug dose–effect relationships (Huang et al., 2017). To date, no specific methods for exploring TCM prescription dose–effect relationships have been developed (Qiu et al., 2018). We will pay close attention to this phenomenon in our future research.

The mechanisms underlying migraine are unresolved; however, it has been demonstrated that inflammation plays crucial roles in headache attacks. Current studies have confirmed that inflammation is present in patients with migraine. It was reported that several major cytokines, such as TNF-α, IL-1β, and IL-6, were elevated in patients during migraine attacks (Yilmaz et al., 2010; Ramachandran, 2018; Edvinsson et al., 2019). The inflammatory response is particularly initiated by CGRP, pituitary adenylate cyclase-activating polypeptide (PACAP), NO, and SP and subsequently causes vasodilatation accompanied by meningeal mast cell degranulation (Irmak, et al., 2019; Cetinkaya et al., 2020; Kilinc et al., 2020). The degranulation of mast cells leads to the release of multiple proinflammatory substances, including enzymes, neurotrophic factors, proinflammatory cytokines, histamine, and serotonin (Koroleva et al., 2019), which activate meningeal nociceptors and induce peripheral and central sensitization (Levy, 2012; Conti et al., 2019). We demonstrated that the degranulation of mast cells in NTG-injected rats was almost 2.5-fold greater than that in control rats, which is consistent with other results (Guo et al., 2021). It was reported that NTG induces an increase in the NF-κB activity and the levels of cytokines, such as TNF-α, IL-6, and IL-1β (Edvinsson, et al., 2019). In our experiments, we also found that the levels of NO, CGRP, and cytokines, including TNF-α, IL-6, and IL-1β, were increased in NTG-treated rats. All these data suggested that NTG exerted a pharmacological effect by inducing neuroinflammation. XZR reduced the degranulation of mast cells as well as the levels of CGRP, SP, NO, and proinflammatory cytokines, including TNF-α, IL-1β, and IL-6, suggesting that XZR improved migraine by relieving the neurogenic inflammatory response.

The augmentation of inflammatory cytokines causes NF-κB dysfunction (Conti, et al., 2019). NF-κB is believed to be related to multiple signaling pathways in headache attacks. Selective inhibition of NF-κB offers a potential therapeutic approach for the treatment of headache (Reuter et al., 2002). Recently, it was discovered that three genes (NF-κBIA, TNFAIP3, and ILR2, closely related to the NF-κB family pathway) were abnormally expressed in chronic migraine patients, suggesting that the suppression of NF-κB activation was critical in resolving the upregulated inflammation and reducing the pain involved in the pathophysiology of the studied chronic migraine patients (Perry et al., 2016). Consistent with these results, we also found that NTG significantly activated the NF-κB signaling pathway, as indicated by the decreased IκB and increased p-IKK and p-NF-κB. XZR showed an inhibitory effect on the NF-κB signaling pathway in rats with NTG-induced migraine, suggesting that XZR could be an optimal therapeutic approach for the treatment of headache.

The activation of NF-κB signaling by NO has been commonly observed. NTG, as a donor of NO, always activates NF-kB phosphorylation and neuroinflammation (Chen et al., 2022). NO is involved in the activation of the dura mater and the subsequent activation of trigeminal fibers and the TNC (Giesen, et al., 2020). The synthesis of NO is catalyzed by nNOS, which can be found in dural mast cells, trigeminal nerve endings, and gasserian ganglion cells (Berger et al., 1994), suggesting its importance in trigeminal pain processing. It was reported that NOS activity was increased in patients with chronic tension-type headache (Sarchielli et al., 2002). A nonselective NOS inhibitor improved headache severity and accompanying symptoms in spontaneous migraine attack (Lassen et al., 1997). NO is involved in nociceptive processing in the central nervous system sensitization of pain pathways, and nNOS inhibition reduces central sensitization (Olesen, 2008). NO has been reported to augment the expression of cyclooxygenase-2 (COX-2), TNF-α, and glutathione-synthesizing enzymes and increase NF-κB activity (Umansky et al., 1998). It was also reported that NOS1-derived NO promoted oxidated low-density lipoprotein (OxLDL) uptake and enhanced the release of proinflammatory cytokines (Roy et al., 2020). In maternal inflammation-induced fetal brain injury, nNOS, NF-κB activation, and proinflammatory cytokine levels were found to be increased (Bandara et al., 2021). Furthermore, NTG was reported to increase the NOS activity and CGRP levels, which might promote inflammatory medium exudation synergistically, leading to the activation of the NF-κB pathway (Yao et al., 2020). However, some reports have suggested that NO has an inhibitory effect on the NF-κB activity. The mechanisms underlying this phenomenon are not clear, but it was supported that NO may be involved in the stabilization of IκBα or the nitrosation of the p50 subunit of NF-κB, leading to a decrease in its DNA-binding affinity (Matthews et al., 1996; delaTorre et al., 1998; delaTorre et al., 1999). Our study confirmed that NTG treatment increased both NO levels and NF-κB activation. L-NAME, a nonselective NOS inhibitor, could decrease p-IKK/IKK and p-NF-κB/NF-κB levels in PC12 cells. These data suggested that NO, produced *via* NOS, could promote the NF-κB signaling pathway. As expected, XZR significantly reduced NO production by inhibiting both the expression and activity of NOS (nNOS and iNOS) and then inhibited the NF-κB signaling pathway. More research is needed to explore the relationship between NOS and NF-κB in XZR-treated migraine.

The trigeminovascular system is important in pain transmission in migraine attacks. Nociceptive transmission originates from the activation and sensitization of first-order trigeminovascular neurons (Ashina, 2020). The second-order trigeminovascular neurons are then activated by ascending nociceptive transmission, which is emitted from the trigeminal ganglion and projected to the brainstem. Ascending nociceptive transmission, in turn, activates and sensitizes third-order trigeminovascular neurons in the thalamus, which subsequently relay the nociceptive transmission to the somatosensory cortex and other cortical areas, ultimately resulting in migraine pain (Ashina, 2020). Our study confirmed that XZR treatment significantly reduced the expression of nNOS, iNOS, and NF-κB signaling pathway proteins in Sp5C cells, suggesting the important role of XZR in the ascending nociceptive transmission pathway.

Nociceptive transmission from the TNC is transmitted to higher brain structures, including the thalamus, brainstem nucleus, rostral ventromedial medulla, and PAG (Goadsby et al., 2009; Castro et al., 2017). In particular, PAG networks have been supposed to have an important role in the pathogenesis of migraine. XZR treatment also reduced the expression of the nNOS, iNOS, and NF-κB signaling pathway proteins in the PAG, suggesting the important role of XZR in the nociceptive transmission descending pathway.

We attempted to provide some substance basis for the pharmacological action of XZR. In the present study, the chemical components of XZR were determined by the UHPLC-LTQ-Orbitrap MS method for the first time. The results showed that coumarins, phenolic acids, flavonoids, phthalides, and terpenoids were the main components of XZR. It was reported that coumarins, such as auraptene, reduced NO production and COX-2, TNF-α, IL-1β, and iNOS expression in RAW 264.7 cells (Hsia et al., 2021) and the hippocampus (Amini-Khoei et al., 2022). Phenolic acids, such as gallic acid, decrease NO levels and exhibit antioxidant and anti-inflammatory effects (Awan et al., 2022). 5-O-Caffeoylshikimic acid suppressed not only the production of NO but also the expression of iNOS, TNF-α, and IL-1β (Lee et al., 2014). It was reported that the main flavonoids from a standardized *S. glabra* flavonoid extract, (-)-epicatechin, astilbin, neoastilbin, isoastilbin, and neoisoastilbin, which were present in the XZR extract, significantly inhibited the secretion of IL-1β, IL-6, NO, and NF-κB p-p65 (Zhao et al., 2020). The above evidence provides a potential role for XZR in anti-inflammatory reactions.

Increasing evidence suggests that iNOS does not show a decisive effect on migraine and that iNOS inhibitors fail to improve migraine (Hoffmann and Goadsby, 2012). Furthermore, it was reported that excessive NO is partly released by nNOS in migraine, and nNOS-specific inhibitors might effectively alleviate migraine (Greco et al., 2015). Therefore, we chose nNOS as a target to screen the effective components by Biacore T200. We revealed that imperatorin and xanthotoxin bound to nNOS (468-616). Xanthotoxin was reported to slow the release of IL-6 and TNF-α in RAW 264.7 cells by inhibiting the NF-κB signaling pathway (Lee et al., 2017). Xanthotoxin also exhibits multiple biological functions, such as the regulation of apoptosis and the proliferation of lymphocytes (Heshmati, 2014). These functions may have a synergistic effect with the functions of compounds that act on NOS/NF-κB in a direct or indirect manner.

## Conclusion

The present study indicated that XZR alleviated NTG-induced migraines. The mechanism might be that XZR downregulated the NO production mediated by NOS, inhibited the NF-κB signaling pathway, and then decreased neurotransmitter and cytokine levels and exhibited an anti-inflammatory reaction. Imperatorin and xanthotoxin might be the effective components of XZR. The findings of this study provide insights into the clinical treatment of migraine with XZR.

## Data Availability

The original contributions presented in the study are included in the article/Supplementary Material; further inquiries can be directed to the corresponding authors.
