# Erratum: Xiongshao Zhitong recipe attenuates nitroglycerin-induced migraine-like behaviors *via* the inhibition of inflammation mediated by nitric oxide synthase

**DOI:** 10.3389/fphar.2022.1021407

**Published:** 2022-09-20

**Authors:** Frontiers Production Office

Due to a production error the incorrect versions of Figures 1, 7 were published. The corrected Figures 1, 7 appear below.

**FIGURE 1:**
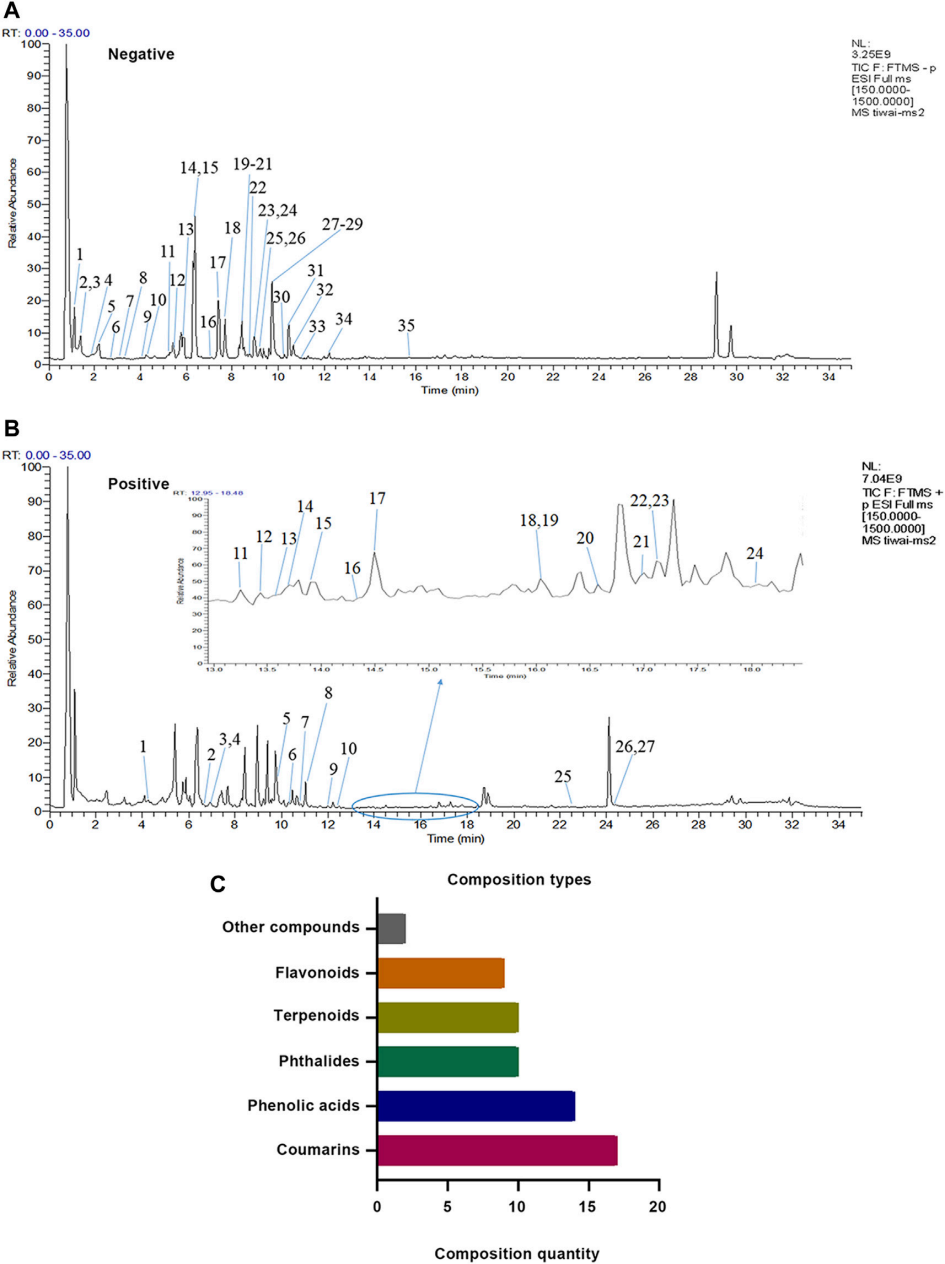
Identification results of themain chemical components of XZR by UHPLC-LTQ-Orbitrap MS. **(A)** Total ion flow diagram of XZR in anion mode (details of Nos. 1–35 are listed in **Supplementary Table S1**). **(B)** Total ion flow diagram of XZR in the positive ion mode (details of Nos. 1–27 are listed in **Supplementary Table S2**). **(C)** Identification of the main components in XZR. XZR, Xiongshao Zhitong Recipe.

**FIGURE 7:**
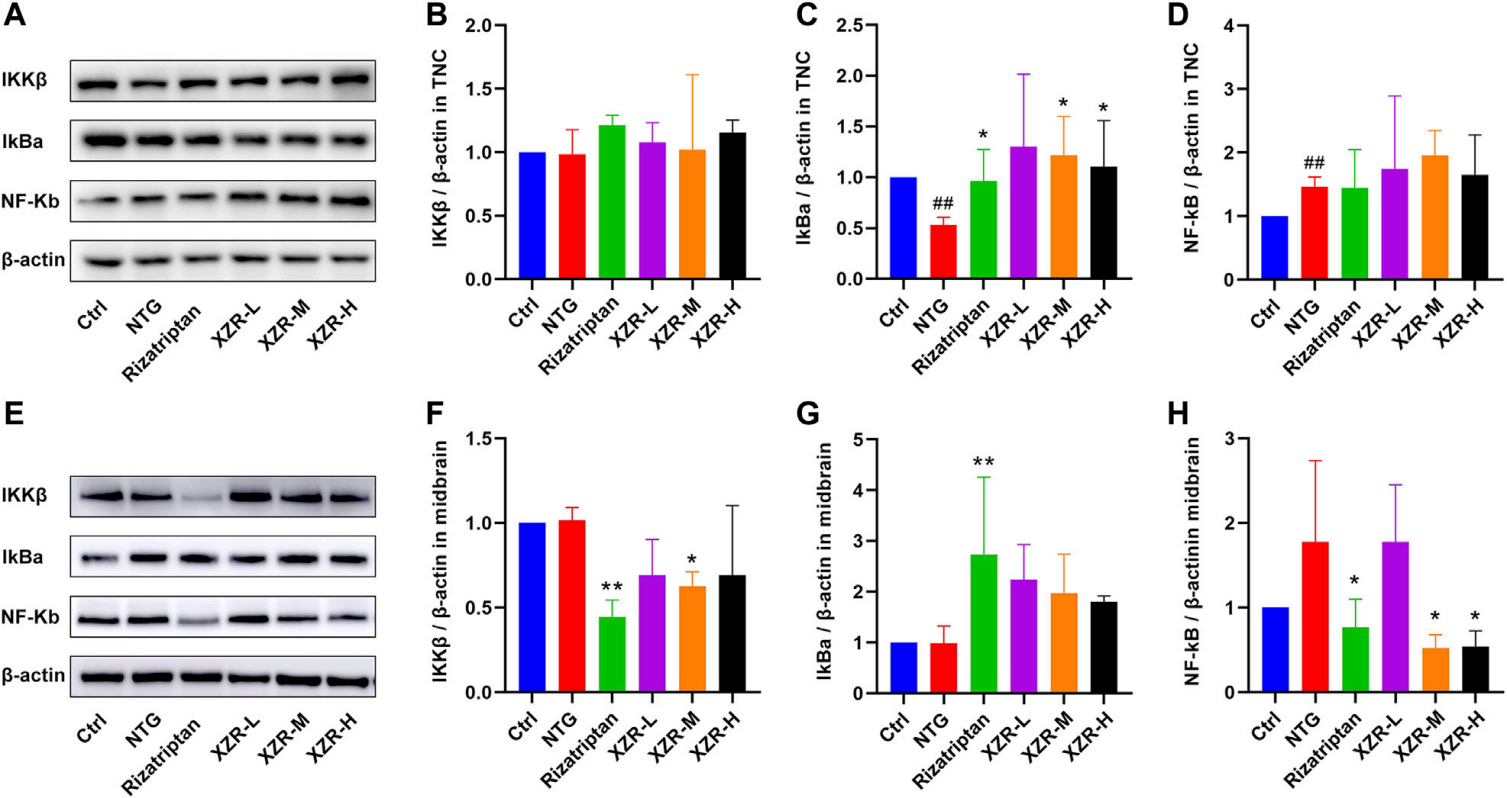
XZR inhibited inflammation via the NF-κB signaling pathway. **(A)** Representative Western blot images of IKKβ, IκBα, and NF-κB expression in the TNC. **(B)** Relative expression of IKKβ in the TNC. **(C)** Relative expression of IκBα in the TNC. **(D)** Relative expression of NF-κB in the TNC. **(E)** Representative Western blot images of IKKβ, IκBα, and NF-κB expression in the midbrain. **(F)** Relative expression of IKKβ in the midbrain. **(G)** Relative expression of IκBα in the midbrain. **(H)** Relative expression of NF-κB in the midbrain. Data are presented as the mean ± standard deviation, *n* = 3–5. ^##^ *p* < 0.01 versus the control group, *p < 0.05, **p < 0.01 versus the NTG group. XZR, Xiongshao Zhitong Recipe; NTG, nitroglycerin.

The publisher apologizes for this mistake. The original version of this article has been updated.

