# Supplementary material for: Xiongshao Zhitong Recipe Attenuates Nitroglycerin-Induced Migraine-Like Behaviors via the Inhibition of Inflammation Mediated by Nitric Oxide Synthase
Source: Front Pharmacol. 2022 Jul 19;13:920201. doi: 10.3389/fphar.2022.920201 (PMC9344691; doi:10.3389/fphar.2022.920201)
Supplement: Supplementary file 1 [file DataSheet1.docx]

Supplementary Material

# Materials and methods

## The Quality of paeoniflorin and salvianolic acid B in XZR

## The determinations of paeoniflorin and salvianolic acid B in the extract of XZR were analyzed by HPLC. The analysis was performed on an Agilent 1100 HPLC system and Agilent ZORBAX SB-C18 column (4.6 mm × 250 mm, 5 μm), with a flow rate of 1.0 mL/min, column temperature of 28°C, and injection volume of 10 μL. The detection wavelength of paeoniflorin was 230 nm, and salvianolic acid B were detected at 286 nm wavelength. The mobile phase was composed of A (0.1% phosphoric acid, v/v) and B (acetonitrile) with a gradient elution: 0–14 min, 14% A; 14.00-14.01 min, 14–23% A; and 14.01-40.00 min, 23% A.

## Gender Evaluation in Behavioral Test

The total 120 SPF adult Sprague-Dawley rats, half male and half female, were randomly divided into six groups, including control group (Ctrl), Nitroglycerin control group (NTG, 10 mg/kg), Rizatriptan group (Rizatriptan, 0.0857 mg/ml), XZR low group (XZR-L, 0.55 g/kg), XZR middle group (XZR-M, 1.09 g/kg) and XZR high group (XZR-H, 2.18 g/kg). Rizatriptan group, XZR-L group, XZR-M group and XZR-H group rats were intragastrically administered with respective drug once per day for 7 consecutive days. All rats, except those in the control group, were subcutaneously injected with NTG 30 minutes after the last treatment. After NTG injected for 30 mins, the frequency of head scratching was measured with a video camera. Briefly, a video camera (DSC-WX9, China) was placed away from the cubicle in positions facing the subject. After NTG injection 30 min later, all rats were acclimatized to the cubicles for 5 min and the scratching behaviors of rats were recorded for 1.5 h.

## Acetic Acid-induced Writhing Test

# Sixty mice were randomly divided into six groups, including control group (distilled water), acetic acid group (distilled water, model group), aspirin treatment group (90 mg/kg, positive drug group), and XZR-L (1.09 g/kg), XZR-M (2.18 g/kg), XZR-H (4.37 g/kg) treatment groups. The mice were orally administered with corresponding drugs for 7 days. One hour after the final drug administration, mice were intraperitoneally injected with 0.6% acetic acid at a dose of 0.1 mL/10 g. The number of contractions was recorded within 15 min after acetic acid injection.

# Supplementary Figures and Tables

# 2.1 Supplementary Table S1

Chromatographic and mass spectral data of the compounds of XZR extract analyzed by UHPLC-LTQ-Orbitrap MS (negative mode)

| NO. | t_R_（min） | MS（*m/z*）  [M-H] or [M+FA-H] | Molecular  formula | MS/MS  （m/z） | Identification | Types |
| --- | --- | --- | --- | --- | --- | --- |
| 1 | 1.14 | 331.0674 | C_13_H_16_O_10_ | 331, 169 | Gallic acid glucopyranosyl | Other (Glycosides) |
| 2 | 1.38 | 169.0130 | C_7_H_6_O_5_ | 169, 125, 107 | Gallic acid | Phenolic acids |
| 3 | 1.43 | 359.0768 | C_16_H_24_O_9_ | 179, 197, 161, 137 | 1-*O*-*β*-*D*-glucopyranosyl paeonisuffrone | Terpenoids |
| 4 | 1.84 | 179.0337 | C_9_H_7_O_4_ | 135, 107, 89 | Caffeic acid | Phenolic acids |
| 5 | 2.11 | 197.0444 | C_9_H_10_O_5_ | 179, 151, 135, 123 | Danshensu | Phenolic acids |
| 6 | 3.06 | 389.1243 | C_16_H_24_O_8_ | 343, 181, 151 | Mudanpioside F | Terpenoids |
| 7 | 3.22 | 421.1328 | C_16_H_24_O_10_ | 375 | Desbenzoyl paeoniflorin | Terpenoids |
| 8 | 2.72 | 407.0591 | C_16_H_26_O_9_ | 361, 119, 89 | 6-*O*-copyranosyl-lacitionlide | Terpenoids |
| 9 | 4.12 | 361.1118 | C_16_H_26_O_9_ | 119, 89 | 6-*O*-*β*-*D*-glucopyranosyl-lactinolide | Terpenoids |
| 10 | 4.24 | 541.1556 | C_23_H_28_O_12_ | 495, 137 | Oxypaeoniflorin | Terpenoids |
| 11 | 5.16 | 447.0926 | C_21_H_20_O_11_ | 401, 269, 161 | Astragalus glycosides | Terpenoids |
| 12 | 5.50 | 335.1570 | C_16_H_16_O_8_ | 179, 135 | 4-*O*-caffeoylshikimic acid | Phenolic acids |
| 13 | 5.86 | 335.1570 | C_16_H_16_O_8_ | 179, 135 | 5-*O*-caffeoylshikimic acid | Phenolic acids |
| 14 | 6.21 | 339.0352 | C_15_H_16_O_9_ | 193, 136, 121 | Smiglanin | Flavonoids |
| 15^*^ | 6.34 | 525.1602 | C_23_H_28_O_11_ | 327, 165, 121 | Paeoniflorin | Terpenoids |
| 16^*^ | 7.10 | 193.0498 | C_10_H_10_O_4_ | 178, 149, 134 | Ferulic acid | Phenolic acids |
| 17 | 7.40 | 449.0459 | C_21_H_22_O_11_ | 303, 285, 151, 107 | Neoastilbin | Flavonoids |
| 18^*^ | 7.74 | 449.0459 | C_21_H_22_O_11_ | 303, 285, 151, 107 | Astilbin | Flavonoids |
| 19 | 8.30 | 449.0459 | C_21_H_22_O_11_ | 303, 285, 151 | Neoisoastilbin | Flavonoids |
| 20 | 8.43 | 449.0459 | C_21_H_22_O_11_ | 303, 285, 151, 107 | Isoastilbin | Flavonoids |
| 21 | 8.52 | 433.1135 | C_21_H_20_O_11_ | 287, 269, 259, 179 | Neo-engeletin | Flavonoids |
| 22 | 8.70 | 433.1135 | C_21_H_20_O_11_ | 287, 269, 259, 179 | Engeletin | Flavonoids |
| 23 | 8.93 | 359.0768 | C_18_H_16_O_8_ | 197, 179, 161 | Rosmarinic acid | Phenolic acids |
| 24 | 8.98 | 313.1443 | C_17_H_14_O_6_ | 269, 203, 159 | Salvianolic acid F | Phenolic acids |
| 25 | 9.03 | 631.1668 | C_30_H_32_O_15_ | 509, 491, 465, 313, 271, 169 | Galloylpaeoniflorin | Terpenoids |
| 26 | 9.11 | 433.1135 | C_21_H_20_O_11_ | 287, 269, 259, 179 | Neoisoengeletin | Flavonoids |
| 27 | 9.54 | 433.1135 | C_21_H_20_O11 | 287, 269, 259, 179 | Isoengeletin | Flavonoids |
| 28 | 9.76 | 537.0621 | C_27_H_22_O_12_ | 339, 295, 313, 179 | Lithospermic acid | Phenolic acids |
| 29^*^ | 9.88 | 717.1454 | C_36_H_30_O_16_ | 339, 321, 295, 279, 185 | Salvianolic acid B | Phenolic acids |
| 30 | 10.26 | 717.1454 | C_36_H_30_O_16_ | 519, 339, 321, 295, 279, 185 | Salvianolic acid E | Phenolic acids |
| 31 | 10.58 | 493.1345 | C_26_H_22_O_10_ | 295, 185, 313, 159, 277 | Salvianolic acid A or isomers | Phenolic acids |
| 32 | 10.62 | 491.0984 | C_26_H_20_O_10_ | 311, 293, 267, 197, 179 | Salvianolic acid C | Phenolic acids |
| 33 | 10.98 | 493.1345 | C_26_H_22_O_10_ | 295, 185, 313, 159, 277 | Salvianolic acid A or isomers | Phenolic acids |
| 34 | 12.20 | 629.1513 | C_30_H_32_O_12_ | 165, 121 | Benzoyl paeoniflorin | Terpenoids |
| 35 | 15.68 | 203.0555 | C_12_H_12_O_3_ | 160, 145 | Senkyunolide B | Phthalides |

Note：^*^Compared with the standard.

**2.2 Supplementary Table S2**

Chromatographic and mass spectral data of the compounds of XZR extract analyzed by UHPLC-LTQ-Orbitrap MS (positive mode)

| NO. | t_R_（min） | MS（*m/z*）  [M+H] | Molecular  formula | MS/MS  （m/z） | Identification | Types |
| --- | --- | --- | --- | --- | --- | --- |
| 1 | 4.22 | 355.0812 | C_16_H_18_O_9_ | 163, 337 | Scopolin | Coumarins |
| 2 | 6.67 | 227.1756 | C_12_H_18_O_4_ | 209, 163 | Senkyunolide J | Phthalides |
| 3 | 7.10 | 225.1484 | C_12_H_16_O_4_ | 207, 189, 179, 165 | Senkyunolide I/Senkyunolide H | Phthalides |
| 4 | 7.12 | 217.0494 | C_12_H_8_O_4_ | 173 | Xanthotoxin isomers | Coumarins |
| 5 | 9.86 | 287.0909 | C_16_H_14_O_5_ | 287, 203, 269, 175 | Pabulenol | Coumarins |
| 6 | 10.38 | 233.0443 | C_12_H_8_O_5_ | 218 | 5-hydroxyxanthotoxin | Coumarins |
| 7 | 10.61 | 305.1019 | C_16_H_16_O_6_ | 203, 159 | Oxypeucedanin hydrate | Coumarins |
| 8 | 11.00 | 317.0993 | C_17_H_16_0_6_ | 233, 299, 218 | Byakangelicol | Coumarins |
| 9 | 11.83 | 189.0909 | C_12_H_12_O_2_ | 171, 128, 115 | E-butylidenephthalide or isomers | Phthalides |
| 10 | 12.35 | 217.0494 | C_12_H_8_O_4_ | 202, 189, 185, 173, 161 | Xanthotoxin | Coumarins |
| 11 | 13.26 | 223.0603 | C_12_H_14_O_4_ | 205, 177 | Senkyunolide D | Phthalides |
| 12 | 13.44 | 287.0909 | C_16_H_14_O_5_ | 203, 269 | Isogospherol | Coumarins |
| 13 | 13.55 | 217.0494 | C_12_H_8_O_4_ | 202, 173 | Bergapten | Coumarins |
| 14 | 13.68 | 247.0599 | C_13_H_10_O_5_ | 231, 217 | Pimpinellin | Coumarins |
| 15 | 13.91 | 189.0909 | C_12_H_12_O_2_ | 171, 153, 128, 115 | Z-butylidenephthalide or isomers | Phthalides |
| 16 | 14.39 | 287.0909 | C_16_H_14_0_5_ | 287, 203, 159, 175, 147 | Oxypeucedanin | Coumarins |
| 17 | 14.49 | 317.0993 | C_17_H_16_O_6_ | 233, 218 | Anhydroisobyakangelicin | Coumarins |
| 18 | 16.04 | 317.0993 | C_17_H_16_O_6_ | 233 | Anhydrobyakangelicin | Coumarins |
| 19 | 16.05 | 287.0909 | C_16_H_14_0_5_ | 287, 203, 187 | Isooxypeucedanin | Coumarins |
| 20 | 16.54 | 271.0962 | C_16_H_14_O_4_ | 203, 187, 69 | Alloisoimperatorin | Coumarins |
| 21 | 16.93 | 193.0495 | C_12_H_16_0_2_ | 175, 147, 137, 119, 105 | Senkyunolide A | Phthalides |
| 22^*^ | 17.09 | 271.0962 | C_16_H_14_O_4_ | 203, 187, 69 | Imperatorin | Coumarins |
| 23 | 17.16 | 193.0495 | C_12_H_16_0_2_ | 175, 147, 137, 119, 105 | Senkyunolide A isomers | Phthalides |
| 24 | 17.97 | 271.0962 | C_16_H_14_O_4_ | 203, 187, 159, 69 | Isoimperatorin | Coumarins |
| 25 | 22.67 | 383.0963 | C_24_H_30_O_4_ | 365, 191 | Senkyunolide P | Phthalides |
| 26 | 24.38 | 381.0790 | C_24_H_28_O_4_ | 191, 173, 163, 145 | Levistolide A | Phthalides |
| 27 | 24.45 | 295.0601 | C_19_H_18_O_3_ | 277, 266, 249, 235 | Tanshinone IIA | Others (Quinones) |
| 18 | 16.04 | 317.0993 | C_17_H_16_O_6_ | 233 | Anhydrobyakangelicin | Coumarins |
| 19 | 16.05 | 287.0909 | C_16_H_14_0_5_ | 287, 203, 187 | Isooxypeucedanin | Coumarins |
| 20 | 16.54 | 271.0962 | C_16_H_14_O_4_ | 203, 187, 69 | Alloisoimperatorin | Coumarins |
| 21 | 16.93 | 193.0495 | C_12_H_16_0_2_ | 175, 147, 137, 119, 105 | Senkyunolide A | Phthalides |
| 22^*^ | 17.09 | 271.0962 | C_16_H_14_O_4_ | 203, 187, 69 | Imperatorin | Coumarins |
| 23 | 17.16 | 193.0495 | C_12_H_16_0_2_ | 175, 147, 137, 119, 105 | Senkyunolide A isomers | Phthalides |
| 24 | 17.97 | 271.0962 | C_16_H_14_O_4_ | 203, 187, 159, 69 | Isoimperatorin | Coumarins |
| 25 | 22.67 | 383.0963 | C_24_H_30_O_4_ | 365, 191 | Senkyunolide P | Phthalides |
| 26 | 24.38 | 381.0790 | C_24_H_28_O_4_ | 191, 173, 163, 145 | Levistolide A | Phthalides |
| 27 | 24.45 | 295.0601 | C_19_H_18_O_3_ | 277, 266, 249, 235 | Tanshinone IIA | Others (Quinones) |

Note: ^*^Compared with the standard.

**2.3 Supplementary Figure S1**


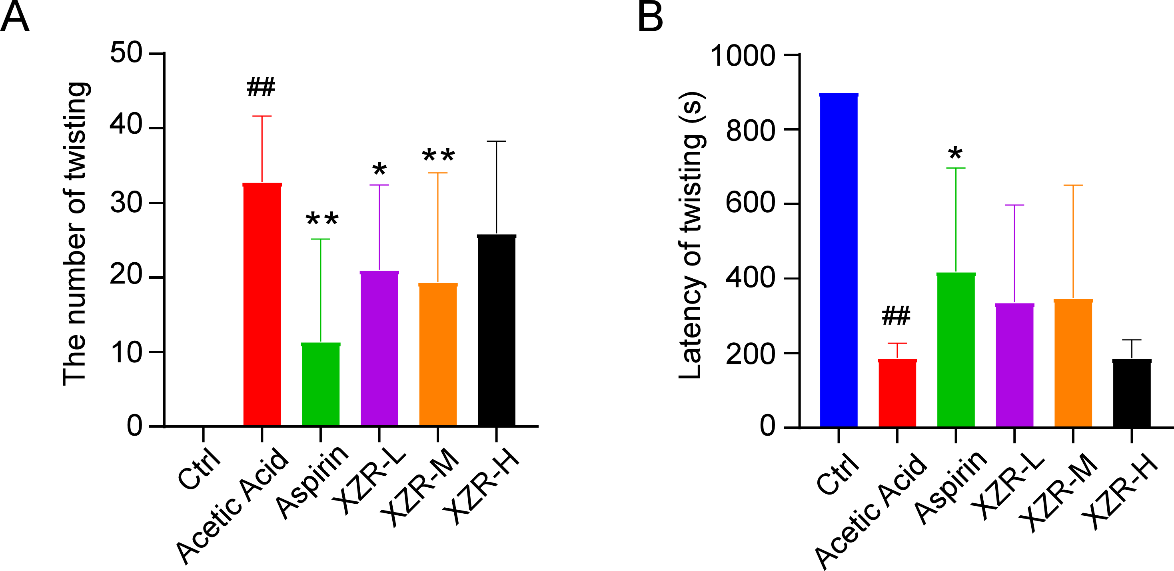


**Supplementary Figure S1.** XZR exhibited an analgesic effect on acetic acid-induced writhing mouse model. (A) The number of twisting. (B) Latency of twisting. Data were presented as mean ± SD. ^##^P<0.01 versus control group, *P<0.05, **P<0.01 versus acetic acid group, n=10.

**2.4**  **Supplementary Table S3**

Chromatographic and mass spectral data of the compounds of the serum analyzed by UHPLC-LTQ-Orbitrap MS (negative mode)

| NO. | t_R_（min） | MS（*m/z*）[M-H] or [M+FA-H] | Molecular  formula | MS/MS  （m/z） | Identification | Types |
| --- | --- | --- | --- | --- | --- | --- |
| 1 | 1.02 | 421.1345 | C_16_H_24_O_10_ | 375, 345, 195, 165 | Desbenzoyl paeoniflorin isomers | Terpenoids |
| 2 | 4.30 | 541.1562/495.1455 | C_23_H_28_O_12_ | 495, 137 | Oxypaeoniflorin | Terpenoids |
| 3^*^ | 6.35 | 525.1602 | C_23_H_28_O_11_ | 327, 121 | Paeoniflorin | Terpenoids |
| 4^*^ | 9.75 | 717.1458 | C_36_H_30_O_16_ | 339, 321, 295, 279, 185 | Salvianolic acid B | Phenolic acids |
| 5 | 13.63 | 537.0621 | C_27_H_22_O_12_ | 339, 295, 313, 179 | Lithospermic acid isomers | Phenolic acids |
| 6 | 15.73 | 203.0702 | C_12_H_12_O_3_ | 160, 145 | Senkyunolide B | Phthalides |

Note：^*^Compared with the standard.

**2.5 Supplementary Table S4**

Chromatographic and mass spectral data of the compounds of the serum analyzed by UHPLC-LTQ-Orbitrap MS (positive mode)

| NO. | t_R_（min） | MS（*m/z*）[M+H]  or [M+Na] | Molecular  formula | MS/MS  （m/z） | Identification | Types |
| --- | --- | --- | --- | --- | --- | --- |
| 1 | 7.12 | 217.0494 | C_12_H_8_O_4_ | 173 | Xanthotoxin isomers | Coumarins |
| 2 | 9.86 | 287.0910 | C_16_H_14_O_5_ | 203, 269 | Pabulenol | Coumarins |
| 3 | 10.60 | 305.1017 | C_16_H_16_O_6_ | 203, 159 | Oxypeucedanin hydrate | Coumarins |
| 4 | 11.00 | 317.0993 | C_17_H_16_O_6_ | 233, 299, 218 | Byakangelicol | Coumarins |
| 5 | 11.59 | 223.0603 | C_12_H_14_O_4_ | 205, 177 | Senkyunolide D isomers | Phthalides |
| 6 | 12.41 | 217.0494 | C_12_H_8_O_4_ | 202,189,185,173 | Xanthotoxin | Coumarins |
| 7 | 13.98 | 189.0908 | C_12_H_12_O_2_ | 171, 153, 128 | *E*-Butylidenephthalide | Phthalides |
| 8 | 14.39 | 287.0910 | C_16_H_14_O_5_ | 203, 175, 159, 147 | Oxypeucedanin | Coumarins |
| 9 | 17.14 | 271.0960 | C_16_H_14_O_4_ | 203, 69 | Imperatorin | Coumarins |

**2.6**  **Supplementary Figure S2**


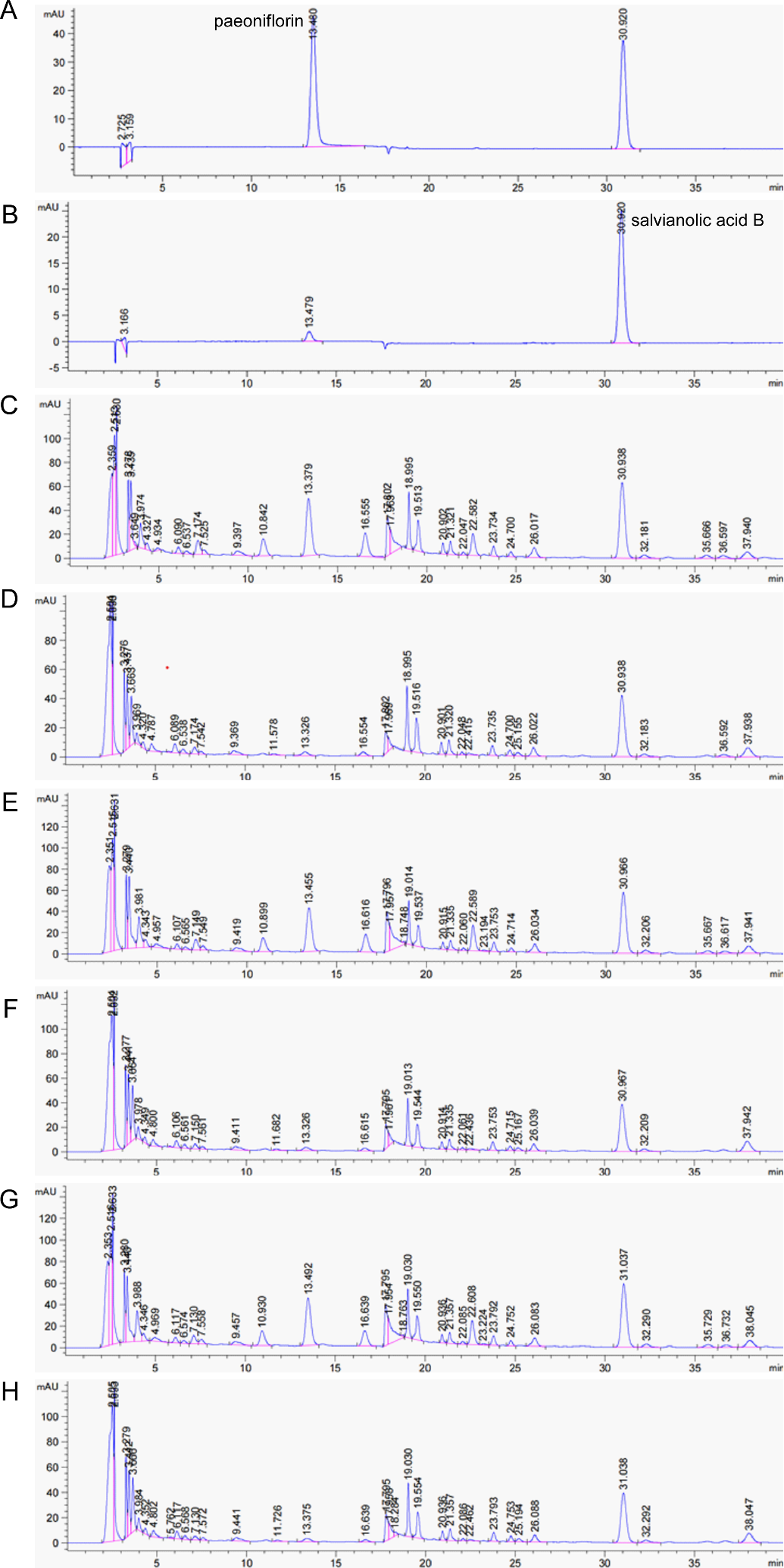


## Supplementary Figure S2. HPLC detection of paeoniflorin and salvianolic acid B in different batches XZR sample. (A) The HPLC figure of paeoniflorin (230nm). (B) The HPLC figure of paeoniflorin and salvianolic acid B (286nm). The HPLC figure of paeoniflorin (C) and salvianolic acid B (D) in XZR sample 1. The HPLC figure of paeoniflorin (E) and salvianolic acid B (F) in XZR sample 2. The HPLC figure of paeoniflorin (G) and salvianolic acid B (H) in XZR sample 3.

**2.7** **Supplementary Table S5**

Transferring rate of paeoniflorin and salvianolic acid B in different samples of XZR

| NO. | Weight  （g） | extraction  yield  （%） | paeoniflorin | |  | salvianolic acid B | |
| --- | --- | --- | --- | --- | --- | --- | --- |
|  |  |  | Transferring rate  (%) | Concentration  (mg/g) |  | Transferring rate  (%) | Concentration  (mg/g) |
| Sample-1 | 0.1997 | 18.20 | 43.48 | 10.87 |  | 53.78 | 16.67 |
| Sample -2 | 0.2003 | 19.40 | 42.51 | 10.63 |  | 51.14 | 15.85 |
| Sample -3 | 0.2000 | 19.40 | 43.34 | 10.83 |  | 54.42 | 16.87 |

**2.8 Supplementary Figure S3**


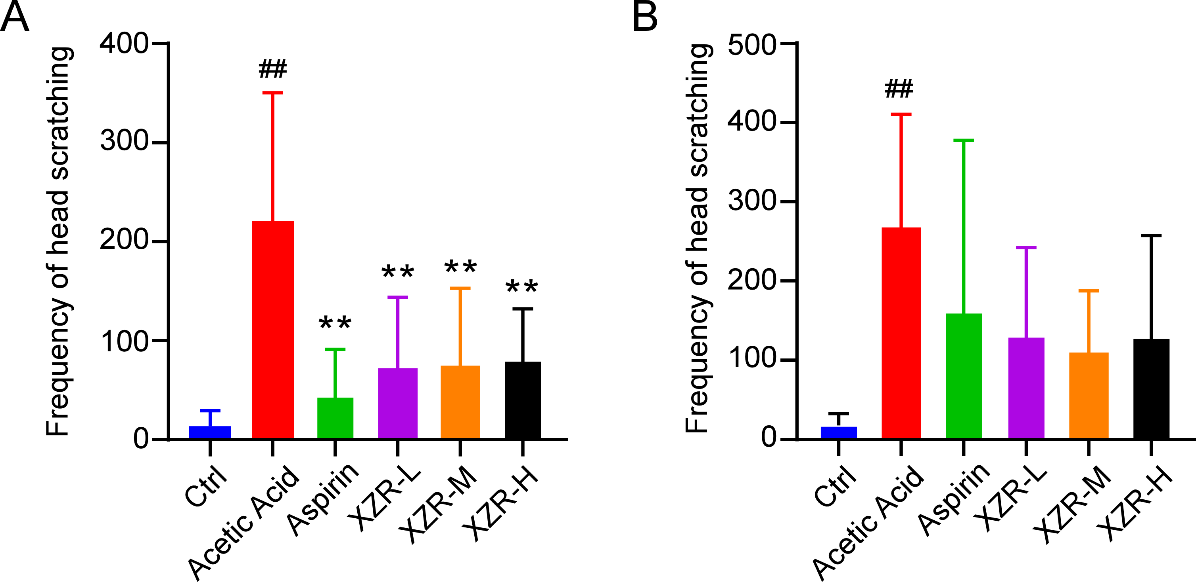


## Supplementary Figure S3. XZR showed better therapeutic effect on male rats than female rats. (A) Frequency of head scratching of male rats. (B) Frequency of head scratching of female rats. Data were presented as mean ± SD. ^#^*P*<0.05, ^##^*P*<0.01 versus control group, **P*<0.05, ***P*<0.01 versus NTG group, n=8-10. NTG: Nitroglycerin.
